# An analysis of WHO FluNet and FluID influenza surveillance data for South East Asia Region, 2015–2023

**DOI:** 10.1371/journal.pone.0341567

**Published:** 2026-02-20

**Authors:** Manish Kakkar, Divita Sharma, Manish Pal, Archisman Mohapatra, Tika Ram Sedai, Francis Yesurajan Inbanathan, Nilesh Buddha, Edwin Ceniza Salvador, Pushpa Ranjan Wijesinghe

**Affiliations:** 1 Infectious Hazards Management Unit, WHO Health Emergencies Programme, WHO Southeast Asia Regional Office, New Delhi, India; 2 Executive Office, Generating Research Insights for Development Council (GRID Council), Noida, Uttar Pradesh, India; The University of Hong Kong, CHINA

## Abstract

**Background:**

Influenza is known to cause seasonal epidemics and recurrent pandemics and demands robust surveillance systems for strain detection and trend monitoring. There are global surveillance systems (FluNet and FluID) to monitor the trends in influenza virus strains and epidemiology. SEAR has been vulnerable to influenza outbreaks and hence, efforts to enhance surveillance have been ongoing. However, there is limited analysis of data trends from influenza surveillance systems from SEAR member states (MS).

**Objectives:**

To describe the virological and epidemiological characteristics of influenza in WHO SEAR and MS therein for the years 2015 till 2023.

**Methods:**

Influenza surveillance data from 2015 to 2023 was extracted for all WHO SEAR MS. This included virological surveillance data from FluNet and epidemiological data from FluID. Descriptive analysis was conducted for the proportionate distribution of Influenza A and B, their subtypes and proportion of ILI and SARI cases. The analysis elicited annual patterns and trends of influenza infections in each MS and across the SEAR region. A multivariable linear regression model was fitted with SARI cases as the outcome against ILI cases, influenza test positivity, country, seasonality and pre-and post- covid period with statistical significance set at p<0.05.

**Results:**

During the reporting period, a total of 5,97,781 specimens were processed in 11 countries. A total of 85,105 (14.2%) specimens were laboratory confirmed influenza positive cases. Two peaks were seen in 2019 and 2021 in almost all the SEAR MS. India (37.4%) had the highest number of confirmed influenza cases followed by Nepal (15.6%) and Bangladesh (10.3%). Influenza A (75.5%) dominated in almost all years and countries. Thailand reported the highest ILI cases (n = 19.4 million; 95.4%), followed by Sri Lanka (n = 0.6 million; 2.9%) and Bhutan (0.8%). Nepal had the highest number of SARI cases (n = 1,03,010; 36.4%), followed by Bangladesh (n = 1,00,772; 35.6%) and Sri Lanka (n = 21,688; 7.7%). In the adjusted model, influenza positivity was associated with higher SARI cases (β = 17.35, p= 0.005), while ILI, seasonality and pandemic period were not.

**Conclusion:**

Enhancing influenza surveillance data can improve epidemic readiness and seasonal vaccination planning. Improving the quality and timeliness of data submissions to FluNet and FluID is crucial, as current data gaps hinder effective decision-making at the regional and global levels. WHO has provided strategic guidance for strengthening the two databases and the Global Influenza Surveillance and Response System (GISRS), urging collaborative and regionally harmonized action including optimizing sentinel site networks and leveraging from COVID-19 for future pandemic preparedness.

## Introduction

Influenza viruses pose a substantial burden on public health [1]. Globally, about 0.29–0.65 million deaths occur every year due to seasonal influenza epidemics [2]. Influenza disease surveillance helps to detect the type of influenza viruses in circulation, monitor seasonal trends, and assess severity of the disease; vaccination and other public health actions could be designed accordingly. The World Health Organization (WHO) provides guidance to conduct influenza sentinel surveillance supplemented by multiple fit-for-purposes mosaic surveillance approach through the Global Influenza Surveillance and Response System (GISRS) [3]. GISRS is a global network launched in 1952 that serves as a mechanism for surveillance, preparedness and response for seasonal, zoonotic and pandemic influenza. Despite years of efforts, the 2009 pandemic due to influenza A(H1N1)pdm 2009 unravelled weaknesses in global, national and local public-health capacities for multi-source influenza surveillance especially for early warning, alert and response. Varying levels of technical capacities, surveillance infrastructure including those for laboratory diagnosis and the need for standardization of surveillance methods were among major challenges identified [4,5]. Subsequently, an attempt was made to strengthen influenza surveillance in WHO Member States (MS) in SEAR along the lines of enhancing core capacities for implementation of International Health Regulations (IHR) (2005), strengthening GISRS network inputs and revising WHO influenza research agenda [6]. To strengthen the global preparedness and response through the GISRS, the global web based, data sharing flatform and the dashboard such as FluNet and FluID of the RespiMART were bolstered. These tools are used for collecting, collating, tracking, analysing and displaying influenza virological data from National Influenza Centre (NIC) laboratories (FluNet) and epidemiological surveillance data from Influenza Like Illness (ILI) and Severe Acute Respiratory Infections (SARI) sentinel surveillance sites (FluID) in WHO Member States [7,8].

WHO’s South East Asia Region (SEAR) accounts for about one quarter of the global population. The region has experienced rapid shifts in social, environmental, and demographic dynamics [9]. The emergence of new ecological niches suggests that the region is likely to remain a hot spot for infectious diseases of epidemic and pandemic significance [10]. SEAR Member States have large, densely populated communities, where many people live in poverty and face inconsistent access to health services. Given above factors combined with the circulation of high-threat pathogens—such as zoonotic influenza—and frequent human-animal interactions, the region remains highly vulnerable not only to seasonal influenza outbreaks but also to zoonotic influenza spill-overs that could be the beginning of emergence of influenza viruses of pandemic potential [9]. Consequently, significant efforts have been made over the years to strengthen influenza surveillance in the region [8]. In this context, WHO’s Regional Office for South East Asia (SEARO) identified ten critical countries to provide financial and technical support for collaborative influenza surveillance through GISRS under the High-Level Implementation Plan II (2018−23) of the Pandemic Influenza Preparedness (PIP) Partnership Contributions (PC) [1,11,12]. The regional office and country-based PIP-PC activity work plans support improving influenza surveillance systems, knowledge sharing and enhancing essential, core laboratory capacities/ needed for timely and appropriate detection, early warning, alert and response to seasonal and zoonotic influenza epidemics and influenza viruses of pandemic potential.

SEAR MS continue to generate and share standardized influenza surveillance data with the global RespiMART data sharing platform (FluNet and FluID) of the GISRS. The integrated analysis of the surveillance (both epidemiological and laboratory) data from these data sharing platforms holds promise for valuable insights into the influenza situation at national, regional levels while contributing to the global level analysis as well. A previous exercise in SEAR that reviewed influenza disease surveillance trends from 2009−2017 showed that the timings of the influenza disease transmission peaks within the region varied from country-to-country but the virus types and subtypes in circulation were similar. This paper concluded by suggesting that timely reporting and sharing of influenza surveillance data could help in improved preparedness for detection of seasonal, zoonotic and pandemic influenza for appropriate response in SEAR MS [13]. The onset of the COVID-19 pandemic in the early 2020s posed significant challenges to existing influenza surveillance systems. This was no exception in the SEAR [14,15]. The COVID −19 pandemic led to shifts in public health resources, healthcare delivery, and surveillance protocols towards response to COVID-19. These together with public Health Social Measures (PHSM) had an propounding impact on conducting influenza surveillance and data reporting to the global platform [15,16]. The extent and nature of these potential disruptions of influenza surveillance data due to the pandemic remains to be fully understood, making comprehensive analysis of surveillance databases even more critical. Against this background, and with a view to sustaining primary objectives of surveilance such as trend analysis for a better understanding of the virological and epidemiological characteristics of influenza in the WHO South-East Asia Region (SEAR) for regional and global health security, we undertook this analysis of data reported to GISRS through FluNet and FluID by all SEAR Member States from 2015 to 2023.

## Materials and methods

### Data source

Influenza sentinel surveillance data reported to GISRS from 2015 to 2023 was extracted from WHO’s RespiMART (FluNet and FluID) for all WHO SEAR MS. Both datasets are publicly available in the public domain through the GISRS [17]. These data sets were further validated with the GISRS team at WHO SEARO for ascertaining any further revisions since extraction of data in May 2024.

FluNet provides virological data from three sources: (a) National Influenza Centres (NICs), (b) from other influenza reference laboratories linked to GISRS, or (c) uploads from the WHO regional database, if any country is unable to upload through above two sources. GISRS linked laboratories test specimens routinely as per the WHO standard guidelines. Those specimens tested positive for influenza are typed as Influenza A or B. Then they are further sub-typed into strains of Influenza A; A(H1N1), A(H1N1)pdm 2009, A(H3N2), A(H5N1), A(H7N9), not subtyped, or un subtypeable. As a standard practice, un-subtypeables are referred to a WHO collaborating centre where they are sub-typed and updated results are then uploaded to the RespiMART by respective MS. Similarly, lineages are determined for Influenza B; Victoria lineage, Yamagata lineage, or lineage not determined. MS submit reports to the FluNet every week. GISRS through WHO SEARO monitors weekly submissions and ensure data are reported for each week. The FluNet, thus, provides weekly virological surveillance data, i.e., weekly number of respiratory specimens that were collected and processed in each country, and the number of those that were tested positive for influenza type A and B, and sub-types. The data is reported and represented in various formats including tables, maps and graphs and are accessible through both WHO GISRS and WHO SEARO web-based dash boards. The FluNet virological data is critical for tracking and determining the circulation of influenza viruses in countries and the region while contributing to meaningfully interpret epidemiological data collected through syndromic Influenza Like Illness (ILI) and Severe Acute Respiratory Infection (SARI) surveillance.

FluID is a component of the RespiMART, the WHO web-based application where FluID collects epidemiological data from syndromic ILI and SARI surveillance and laboratory confirmed influenza cases into a single global database. Data is exported from the national influenza programmes or focal points for influenza surveillance in SEAR MS, weekly or is uploaded from the regional office. It has both qualitative and quantitative data. Qualitative assessments include geographic spread, trend, intensity of transmission, and impact of influenza on health care systems. The sources of quantitative data are the sentinel surveillance sites which report on the numbers of cases enrolled from Influenza-like Illnesses (ILI), Severe Acute Respiratory Infection (SARI) and/or Acute Respiratory Infections (ARI). These data are disaggregated by standard age groups and include denominators such as total number of facility/outpatient visits for ILI and the total number of in-patients for SARI. The database also reports on the number of ILI and SARI cases (from which specimens were collected) attributable to Influenza, and on the number of deaths among SARI cases. In FluID, ILI is defined as an acute respiratory infection with (a) measured fever of ≥ 38 C°, and (b) cough with (c) onset within the last 10 days while SARI is defined as an acute respiratory infection with (a) history of fever or measured fever of ≥ 38 C°, and (b) cough, with (c) onset within the last 10 days and (d) requiring hospitalization [18].

### Data extraction and analysis

Data was exported from the WHO’s RespiMART database for the reporting period 2015−2023 to a Microsoft Excel spreadsheet (version 2601, build 19628.20204) for data management and analysis. Weekly surveillance data were aggregated at the annual level and stratified by member states, hemisphere and influenza transmission zones for descriptive analysis. Rates were calculated separately for each member state and then data was aggregated to arrive at regional estimates, without population standardization given the surveillance-based nature of the data. Zero counts for subtypes or periods without cases were retained in the dataset and included in proportion calculations as zero values. Frequencies and proportions were calculated for laboratory confirmed influenza cases per total samples processed, absolute number of ILI and SARI cases, ILI cases per 1,000 outpatients, SARI cases per 100 in-patients and SARI case fatality ratio. Seasonal distribution of influenza types and subtypes/lineages was assessed for each MS to explore trends over time. Visual representations including tables and graphs were generated to illustrate trends across all years and for each MS. A multivariable linear regression model was fitted with SARI cases as the outcome. Predictors included ILI cases, the proportion of influenza-positive samples, seasonal quarter, pandemic period (pre-COVID-19, COVID-19, post-COVID-19), country fixed effects for WHO South-East Asia Region Member States, and physician density. The results were considered significant if the p-value was less than 0.05. We did not report on data/ indicators that were implausible due to possible data quality concerns.

### Ethics

This study did not collect any primary data from human participants and used pre-existing, anonymized open-source data that can be downloaded online without login; the requirement for ethical approval of this study and securing consent from the participants was hence not required. On the other hand, as ILI/SARI surveillance is a routinely conducted activity, none of the SEAR MS obtains ethical approval at the country level. The datasets for FluNet and FluID used for analysis are attached in the S1 and S2 File.

## Results

All member states from WHO SEAR reported virological data to FluNet from 2015 to 2023 except for Myanmar and Timor-Leste which reported data from 2016 and Democratic People’s Republic of Korea (DPRK) which reported data from 2017. Epidemiological (FluID) data was available for nine SEAR MS, i.e., Bangladesh, Bhutan, DPRK, Indonesia, Maldives, Nepal, Sri Lanka, Thailand and Timor-Leste. Only Bhutan and Indonesia provided data for the entire reporting period whereas data for other countries was incomplete. Although an inconsistent pattern in reporting was observed, the numbers of ILI cases were provided by all the nine MS reporting epidemiological data. Data on SARI deaths were available from only four SEAR MS namely, Bangladesh, Bhutan, Maldives and Nepal. We excluded data for DPRK and Timor Leste for estimating SARI cases per 100 inpatients as there were errors in reporting.

### Influenza virus circulation in WHO SEAR: virological data trends (Source: FluNet)

The total number of specimens processed annually based on FluNet data fluctuated between 44,763 in 2015–83,588 in 2023. From 2015 to 2019, the number of specimens processed increased by 91.9% (n = 44,763 in 2015; n = 85,904 in 2019). Across all the years, the total number of samples processed was 5,97,781 of which the maximum number of samples were processed in the year 2021 itself (n = 1,48,664, 24.9%). Between 2015 and 2023, India (52.2%) contributed maximally to the total specimens processed followed by Bangladesh (13.8%) and Nepal (8.2%).

Of the 5,97,781 samples processed, 85,105 samples (14.2%) were confirmed as laboratory ‘positive’ cases of Influenza. Between 2015 and 2019, the percentage of influenza positive cases ranged between 20.4% and 25.6% across the region (aggregated for all member states). In 2020, this figure dropped sharply to 9.7%, followed by a further decline to 5% during 2021–2022. However, there was an increase in cases in 2023, rising back to 14.2%. Between 2015 and 2023, Influenza virus type A accounted for 75.5% cases and Influenza virus type B for 24.5%. Influenza type A was the predominantly circulating influenza virus in all the years except in 2016 when Influenza type B dominated (Table 1).

**Table 1:** Laboratory confirmed positive cases of influenza viruses in circulation by type and subtype/lineage in WHO SEAR, 2015-2023, n(%).

Influenza virus types/subtypes and lineages	Year									
	2015^a^	2016^b^	2017	2018	2019	2020	2021	2022	2023	Total
**Type A (Total)**	**9804** **(90.9)**	**2978** **(48.0)**	**9898** **(82.3)**	**5702** **(77.1)**	**15272** **(75.8)**	**2958** **(89.7)**	**5169** **(63.8)**	**4721** **(90.4)**	**7736** **(64.9)**	**64238** **(75.5)**
*A(H1N1)pdm2009*	7729 (71.7)	745 (12.0)	6456 (53.7)	3644 (49.3)	9876 (49.0)	1908 (57.9)	657 (8.1)	1971 (37.8)	2889 (24.2)	**35875** **(42.2)**
*A(H1)*	–	–	–	–	–	–	–	–	–	**–**
*A(H3)*	1144 (10.6)	2200 (35.5)	2094 (17.4)	1875 (25.3)	5149 (25.6)	1029 (31.2)	4487 (55.3)	2687 (51.5)	4691 (39.3)	**25356** **(29.8)**
*A(H5)*	1 (0.01)	–	–	–	1 (0.005)	–	–	–	–	**2 (0.002)**
*A(H7N9)*	–	–	–	–	–	–	–	4 (0.001)	–	**4 (0.01)**
*A(not subtyped)*	929 (8.6)	18 (0.3)	1336 (11.1)	83 (1.1)	236 (1.2)	21 (0.6)	25 (0.3)	52 (1.0)	156 (1.3)	**2856** **(3.4)**
*A(not subtypeable)*	–	–	–	–	6 (0.03)	–	–	7 (0.001)	–	**13 (0.02)**
*A(other subtype)*	1 (0.01)	15 (0.2)	12 (0.1)	100 (1.4)	4 (0.02)	–	–	–	–	**132 (0.2)**
**Type B (Total)**	**979** **(9.1)**	**3224** **(52.0)**	**2133** **(17.7)**	**1696** **(22.9)**	**4866** **(24.2)**	**340** **(10.3)**	**2938** **(36.2)**	**500** **(9.6)**	**4191** **(35.1)**	**20867** **(24.5)**
*B(Victoria lineage)*	7 (0.1)	549 (8.9)	280 (2.3)	150 (2.0)	2354 (11.7)	178 (5.4)	2373 (29.3)	340 (6.5)	3223 (27.01)	**9454** **(11.1)**
*B(Yamagata lineage)*	252 (2.3)	464 (7.5)	446 (3.7)	642 (8.7)	229 (1.1)	6 (0.2)	3 (0.04)	–	–	**2042** **(2.4)**
*B(lineage not determined)*	720 (6.7)	2211 (35.6)	1407 (11.7)	904 (12.2)	2283 (11.3)	156 (4.7)	562 (6.9)	160 (3.1)	968 (8.1)	**9371** **(11.0)**
**Total Positive**	**10783**	**6202**	**12031**	**7398**	**20138**	**3298**	**8107**	**5221**	**11927**	**85105**

Data given in n (%).

^a^Data available for all WHO SEAR member states except DPRK, Myanmar and Timor- Leste.

^b^Data available for all WHO SEAR member states except DPR Korea.

**Fig 1:**
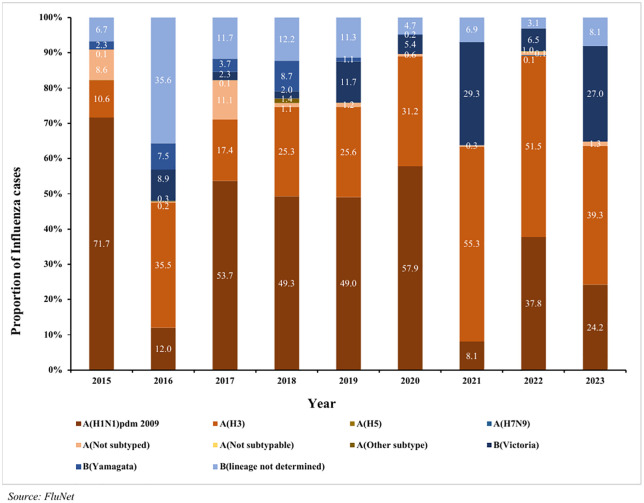
Proportionate distribution of influenza viruses by subtypes in WHO SEAR, 2015-2023.

The dominant subtype in Influenza Type A was A(H1N1)pdm 2009 in most of the years except in 2016, 2021, 2022 and 2023 when A(H3) was the dominant subtype. Among the influenza Type B, B(lineage not determined) was dominant between 2015 and 2018 and B(Victoria) from 2019 and 2023. Overall, among the Type B strains, a substantial proportion was attributed to ‘lineage not determined’ in each year except from 2021 till 2023 (Fig 1).

India (37.4%) had the highest proportion of influenza cases followed by Nepal (15.6%) and Bangladesh (10.3%). Two distinct peaks in Influenza cases were observed in 2019 and 2021 across most SEAR member states. In contrast, a notable declined occurred in 2020 in nearly all member states except Indonesia. This downward trend persisted into 2022 for most countries, with the exception for Bhutan, Indonesia, Sri Lanka, Thailand and Timor Leste. Influenza cases per million population demonstrated heterogeneity between Member States. While 2019 represented a higher count for several countries, increases in 2021 were observed in only a limited number of Member States (Maldives and Timor-Leste) rather than across the region (S3 Fig).

**Fig 2:**
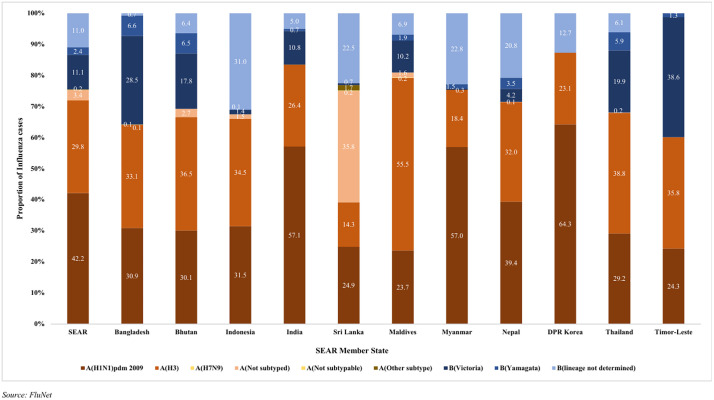
Proportionate distribution of influenza viruses by subtypes and lineages in WHO SEAR Member States, 2015-2023.

Influenza A was the more commonly occurring type than Influenza B in SEAR and in respective MS. In 2016, Influenza type B was seen in proportions higher than that for Influenza A in all the MS (except in Maldives and Thailand). Variation was also seen in the type of Influenza virus subtypes circulating in different MS. Of all the Type A influenza cases, A(H1N1)pdm 2009 was overall dominant (42%) except in Maldives where A(H3) dominated accounting for 55.5% of cases. Of the Influenza B types, B(Victoria) lineage and B(lineage not determined) were in co-circulation in SEAR. B(Victoria) lineage dominated in most of the MS except Indonesia, DPRK, Myanmar, Nepal and Sri Lanka where B(lineage not determined) was predominant. B(Yamagata) lineage was seen to be less in circulation and was only reported in Bangladesh, Bhutan, India, Nepal and Thailand (Fig 2).

#### Influenza virus circulation according to the hemispheres.

GISRS has defined two hemispheres for influenza virus circulation. Categorization of MS in SEAR by hemispheres is as follows; i.e., Northern (Bangladesh, Bhutan, DPR Korea, India, Maldives, Myanmar, Nepal, Sri-Lanka, and Thailand) and Southern (Indonesia and Timor-Leste) hemispheres. In the Northern hemisphere, circulation of Influenza virus type A dominated over that of Type B in almost all the years except in 2016. In the Southern hemisphere, the circulation of Influenza type A and type B viruses was nearly similar, except in 2020 when Influenza type A viruses dominated.

Considering the subtypes of Influenza A viruses, A(H1N1)pdm 2009 dominated in the Northern hemisphere whereas in the Southern hemisphere virus type A(H3) dominated. There was a sudden peak of Influenza A(H3) in 2016 and 2021. Influenza A(H5), A(H7N9), A(not subtyped) and A(not sub-typeable) showed very low or negligible circulation throughout the years.

In terms of Influenza type B lineages, B(Victoria) dominated in 2020, 2021, 2022, and 2023 in both hemispheres, B(lineage not determined) was predominant from 2015 to 2019 in both hemispheres and B(Yamagata) lineage circulated in the northern hemisphere from 2015 to 2019. However, from 2019 onwards, the prevalence of B(Yamagata) lineage decreased significantly in both hemispheres (S4 Fig).

#### Influenza virus circulation according to the WHO influenza transmission zones (ITZ).

**Fig 3:**
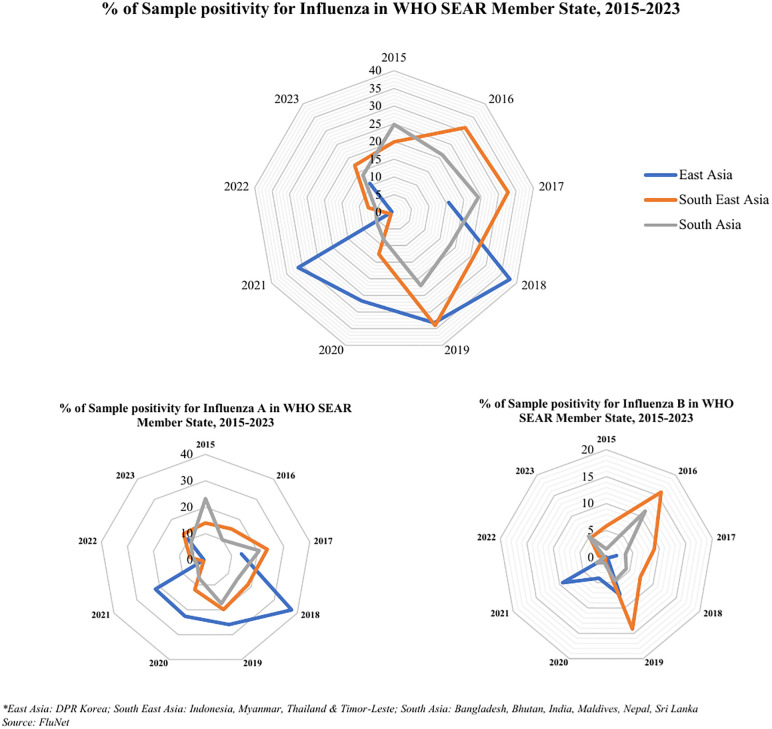
% of Sample positivity for Influenza according to the Influenza Transmission Zones in WHO SEAR Member States, 2015–2023.

In SEAR, influenza virus circulation varied across three WHO defined transmission zones; East Asia, Southeast Asia and South Asia. Influenza A virus dominated in all the three transmission zones. In East Asia, Influenza virus subtype A(H1N1)pdm 2009, dominated most years, with only Influenza type B(lineage not determined) in circulation. Southeast Asia saw dominance of A(H3) among Influenza A sub-types, except for 2017–2019. For B lineages, Influenza B(lineage not determined) dominated from 2015 to 2018, followed by B(Victoria) lineage from 2019 to 2023. In South Asia, mostly Influenza sub-types A(H1N1)pdm 2009 was in circulation with co-circulation of B(Victoria) lineage and B(lineage not determined). B(Yamagata) lineage was lesser in circulation, only during the years 2015–2018. Across SEAR, Influenza B(lineage not determined) was more common than B(Victoria) lineage, prevailing in all four transmission zones (Fig 3). Graphs representing circulation of influenza viruses in transmission zones are provided in S5 Fig.

### Bangladesh

Influenza A(H1N1)pdm 2009 predominated in almost all the years except in 2016 and 2022. It was seen more in the months of April- September (Summer and Monsoon) throughout the years except 2020 where it was also seen during January to March (Winter/ Spring). Co-circulations with Influenza A(H1N1)pdm 2009 with A(H3) was seen during January to March (Winter/ Spring) from 2015 in almost all the years except in 2022, with B(Victoria) lineage in most of the years except in 2015, 2020 and 2022 and B(Yamagata) lineage from 2015−19. It was observed that there was an increase in trend of B(Yamagata) lineage till 2018 and B(Victoria)lineage in almost all the years except in 2015, 2018 and 2020. This trend was observed mainly in the months from April to September (Summer and Monsoon). However, in 2020, most of the cases were in the months starting from September to December; A(H3) was predominant. In 2021, Influenza B(Victoria lineage) activity was seen during the first half of the year with A(H1N1)pdm 2009 activity increasing in the later part of the year.

### Bhutan

The data showed high activity of sub type A(H1N1)pdm 2009 in 2015, 2017, 2019 and 2020 and then decreased thereafter. A(H3) subtype showed varying activity across different years, with peaks in some years such as 2016, 2022 and 2023. The total influenza B cases also fluctuated across years, with notable peaks in 2015, 2019, and 2021. B(Victoria) lineage and B(lineage not determined) contributed to the influenza B activity, with varying levels of activity in different years. Influenza A virus was mostly in circulation during the months of January to March and Influenza B virus was mostly seen in later parts of the year.

### Democratic People’s Republic of Korea (DPRK)

DPRK started reporting the virological data in 2017 with influenza cases of A(H1N1)pdm 2009, A(H3) and B(lineage not determined). A(H1N1)pdm 2009 showed varying activity levels across the years, with maximum number of cases in 2022. A(H3) subtype also exhibited fluctuating activity, with noticeable peaks in 2019 and during the months of January to March (Winter/ Spring) in 2023. In 2018, there was a peak of A(H1N1)pdm 2009 cases from January to March (Winter/ Spring) but continued to decline for the rest of the year. For the rest of the years till 2021, there was a consistent trend of A(H1N1)pdm 2009 and A(H3) with B(lineage not determined) in co-circulation. There were no reported cases for B(Victoria), B(lineage not determined), and B(Yamagata) throughout the years. A(H1N1)pdm 2009 was seen more in the later parts of the year while sub-type A(H3) was seen more during January- March (Winter/ Spring). B(lineage not determined) showed varied activity from 2017–2021 and showed little activity in the subsequent years.

### India

In 2015, India reported high activity of A(H1N1)pdm 2009, A(H3) and B(lineage not determined). Cases of A(H1N1)pdm 2009 was seen throughout the reporting years 2015–2023. The cases were usually high during the first half of the year and gradually declined towards the other half of the year except in 2018 and 2023, where the cases were also observed in the second half. During January-March 2015, 2016 and 2019, there was a large number of A(H1N1)pdm 2009 positive specimens resulting in a substantially higher peak in influenza positives than in any other year. However, in 2018, 2022 and 2023 a reverse trend was observed where cases were higher in the months July-December. A high peak of A(H3) was observed in January-March (Winter/ Spring) 2016, 2021, 2022 and 2023. A(H3) sub-type circulation was also observed in the later part of the years 2016, 2020, 2021 and 2022. The activity of B(lineage not determined) was reported in inter-seasonal periods. In 2017, 2018, 2020, 2022 and 2023 the cases were mostly seen in the first half of the year and then gradually declined while in the year 2015, 2016, 2019 and 2021, B(lineage not determined) cases were mostly seen in the second half of the year. The cases of B(Yamagata) lineage emerged in 2016 and then it was seen in circulation in the years 2018 and 2019. B(Yamagata) lineage circulation was low to negligible post 2019, and B(Victoria)lineage continued to be in circulation from 2016 to 2023, throughout almost all the seasons.

### Indonesia

In the years 2016, 2018, and 2022, we observed a significant peak in A(H1N1)pdm 2009 cases during the first half of the year. Conversely, in 2015, 2017, 2019, 2022, and 2023, this peak was noted in the second half of the year. There were no reported A(H1N1)pdm 2009 cases in 2020 and 2022. A(H3) sub-type was predominantly observed in the first half of each year, except for 2019, 2020, and 2021. A(not subtyped) strains circulated in 2018, with minimal activity in July to September 2022 and 2023. Influenza B activity was observed throughout all years except for 2020. B(lineage not determined) was the dominant, especially evident during the mid-year period from April to September, except for 2020, 2021, and 2022. B(Victoria) lineage circulation was notable in recent years, particularly in 2021, 2022, and 2023. In the first half of 2021, B(Victoria) lineage was prominent, comprising 38% of cases from January to March and 50% from April to September. Conversely, B(Yamagata) lineage circulation was observed solely in 2020.

### Maldives

In 2017, a large number of A(H1N1)pdm 2009 samples were reported, with the outbreak starting in February and peaking in March. A second peak was observed during October to December 2018 followed by January to March 2019. Every year, A(H1N1)pdm 2009 circulation was seen in the months of January to March, except in 2015, 2018 and 2023. A peak of Influenza A(H3) activity was observed during the April month of the years 2016, 2018, 2022 and 2023. In 2021, the peak was seen in the months of July to December and in 2022, A(H3) peak was seen in the months of April to September. Then a continuous fall in cases was seen till September 2020 and then again, a peak was observed during October-December 2021. Influenza B activity increased in September and the virus continued to circulate until the end of 2019. In the recent years (from 2021–2023), co-circulation of A(H3) sub-type with only B(Victoria) lineage was seen.

### Myanmar

Myanmar started reporting from 2016. Influenza B(lineage not determined), Influenza A(H1N1)pdm 2009, and A(H3) were the dominant subtypes. Between 2016 and 2019, A(H1N1)pdm 2009 accounted for the majority of influenza-positive specimens. Notably, in 2017 (throughout all months) and from January to September 2023, Influenza A(H1N1)pdm 2009 prevailed over other subtypes. In recent years, the co-circulation of Influenza B lineage was minimal or negligible.

### Nepal

In 2015, influenza A(H1N1)pdm 2009 sub-types and, among influenza B types, B(Yamagata)lineage was predominant, with both peaking from January to March (Winter/ Spring). Following this, in each subsequent year, there was a noticeable increase in the trend of B(lineage not determined) alongside A(H1N1)pdm 2009 and A(H3) sub-types. From 2015 to 2021, a peak in influenza activity during January to March (Winter/ Spring) was primarily attributed to influenza A(H1N1)pdm 2009, while a later peak in July to September was mainly due to A(H3) sub-type circulation. Influenza A(H1N1)pdm 2009 was observed to circulate throughout the year except in 2020, 2021, and 2022. Notably, from April 2020 to March 2022, there was no circulation of Influenza A(H1N1)pdm 2009.

### Sri Lanka

A consistent co-circulation of Influenza A(H1N1)pdm 2009, A(H3), A(not subtyped) and B(lineage not determined) was seen from 2015–2019 and then again in 2022–2023. The activity of A(H1N1)pdm 2009 varied across the years. It peaked in 2015, 2016, and 2018, with notable activity in the first half of each year. A(H3) sub-type demonstrated varying levels of activity, peaking in 2015, 2016, and 2019, with sporadic activity in other years. Influenza A(not subtyped) and B(lineage not determined) was seen throughout the reporting period with maximum activity of B(lineage not determined) seen in 2016 and 2020. A(H1), A(H5) subtypes and B(Yamagata)lineage showed minimal activity throughout the observed period, with no significant peaks detected.

### Thailand

In Thailand, A(H1N1)pdm 2009 showed sustained circulation throughout most months, except for 2020 and 2023 where no activity was seen. Influenza A(H3) was seen consistently active in the first half of each year except in 2019. B(Victoria) lineage showed activity across all years, with varying intensity while B(lineage not determined) displayed fluctuating activity levels across the years and months. B(Yamagata) lineage was only prominent in 2015, 2016, and 2018. Other subtypes like A(H1), A(H5), and A(not subtyped) showed minimal to no activity throughout the observed period. From 2016 to 2021, there was a consistent trend of increasing B (Victoria) lineage and B (lineage not determined) activity alongside influenza A(H1N1)pdm 2009 and A(H3) sub-types. In these years, January to March (Winter/ Spring) typically saw transmission peaks primarily due to influenza A(H1N1)pdm 2009 sub-type, while July to September (Monsoon) exhibited peaks mainly attributed to influenza A(H3).

### Timor-Leste

Virological data was reported from the year 2017. Except for the year 2021, co-circulation of A(H1N1)pdm 2009, A(H3) sub-types and B(Victoria) lineage was seen. A(H3) showed the highest activity except in the years 2018, 2019 and 2023. It was mostly seen in the months of January to March but in the year 2021 it was present throughout the year without any other subtypes in circulation. Minimal activity of A(H1N1)pdm 2009 sub-type was mostly seen during the first half of the year, with a notable increase only in 2022. B(Victoria) lineage was observed from 2018–2023, except for 2021 and no significant seasonal pattern was observed. Minimal or no activity was observed for A(H1), A(H5) sub-types, B(Yamagata) lineage and B(lineage not determined).

Proportion of influenza viruses by subtypes in WHO SEAR member states from 2015–2023 is represented in S6 Fig.

### Epidemiological data trends for influenza in SEAR (Source: FluID)

Data for FluID was contributed by nine SEAR MS only. Bhutan, Indonesia and Thailand provided weekly data since 2015 but none of the MS provided weekly data consistently. The numbers and reporting patterns had ‘nose-dived’ from 2020 and onwards for ILI cases and increased for SARI cases. From the data available, it was observed that most member states exhibited mixed seasonality patterns for ILI and SARI cases.

In the adjusted regression model with country fixed effects, the proportion of influenza-positive samples was significantly associated with higher SARI cases (β = 17.35, p = 0.005), while ILI cases showed no association (p = 0.955). Seasonality, COVID pandemic, and physician density were not significantly associated with SARI cases. Compared with the reference country, all included countries showed significantly lower SARI case counts (p < 0.001 for all). The model demonstrated strong explanatory power (adjusted R² = 0.72, p < 0.001) (Table 2).

**Table 2:** Predictors of Severe Acute Respiratory Infection (SARI) in WHO SEAR, 2015-2023.

Overall model (adj. R^2^ = 75.4%) Y= SARI cases	b (S.E.)
ILI cases	-.00006 (.00108)
Proportion of influenza positive cases	17.35 (6.06)**
**Month (Ref: Apr-Jun)**	
January-March	295.35 (210.37)
July-September	56.44 (216.68)
October-December	114.55 (211.92)
**Time period (Ref: pre-COVID (2015-2019))**	
COVID (2020-2021)	350.92 (232.28)
Post-COVID (2022-2023)	-4.85 (353.52)
**Country (Ref: Nepal)**	
Bangladesh	-1956.35 (329.32)***
Bhutan	-3811 (368.14)***
Indonesia	-4024.29 (675.20)***
Maldives	-4402.38 (526.59)***
Sri Lanka	-2696.76 (526.59)***
Thailand	-4384.76 (535.65)***

*p < 0.05, **p < 0.01, ***p < 0.001.

The total number of ILI cases were high in 2015 and 2017−19, and showed a dip from 2020 to 2023. Overall, a total of 20.3 million ILI cases were reported from SEAR. Thailand (n = 19.4 million; 95.4%) reported the highest number of cases followed by Sri Lanka (n = 0.6 million; 2.9%) and Bhutan (n = 0.2 million; 0.8%). Out of total SARI cases (n = 2,83,330) reported in SEAR, Nepal (n = 1,03,010; 36.4%) had the highest number of SARI cases followed by Bangladesh (n = 1,00,772; 35.6%) and Sri Lanka (n = 21,688; 7.7%).

**Fig 4:**
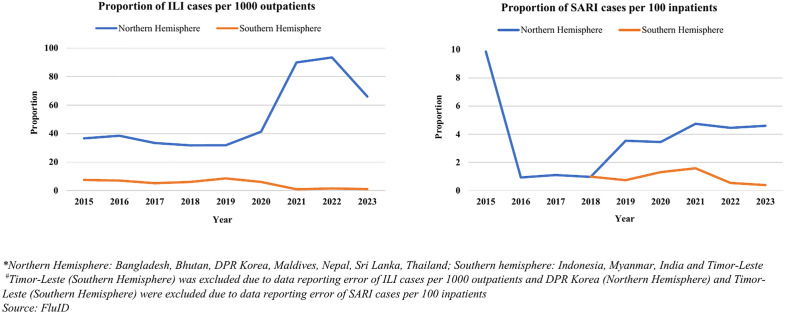
Proportion of ILI cases per 1000 outpatients and SARI cases per 100 inpatients in WHO Northern and Southern Hemisphere, 2015-2023.

**Fig 5:**
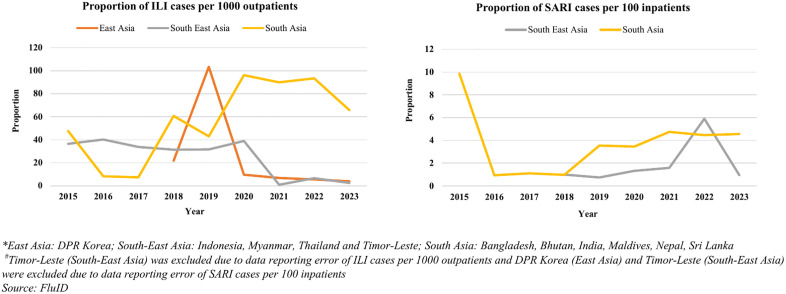
Proportion of ILI cases per 1000 outpatients and SARI cases per 100 inpatients in WHO Influenza Transmission Zones, 2015-2023.

The number of ILI cases per 1000 outpatients were in the range of 31–40 from 2015–2021. These showed a hike in 2022 with 58 cases and in 2023 with 44 cases per 1000 outpatients. 2015 was apparently an outlier for reporting on the number of SARI cases per 100 inpatients (21/100 inpatients). SARI cases in terms of absolute numbers showed a gradual upward trend from 2016 till 2019 followed by a decline in 2020. The number of SARI cases subsequently showed an increased trend from 2021–23. However, SARI cases per 100 inpatients remained similar from 2016 to 2018, increased 3.7 folds from 1/100 in 2018–4/100 in 2019 and ranged between 3–5 cases per 100 inpatients in the subsequent years (S7 Fig). The variations of proportion of ILI cases per 1000 outpatients and proportion of SARI cases per 100 inpatients were also seen in both the hemispheres and ITZ. The Northern Hemisphere consistently exhibited higher ILI cases throughout, with a peak observed in 2022 at 93 cases per 1000 outpatients, while the Southern Hemisphere generally had lower levels, peaking at 9 cases per 1000 outpatients in 2019. However, both hemispheres experienced fluctuations, with the Northern Hemisphere showing a significant increase from 2020 to 2022, while the Southern Hemisphere saw a decline in 2021 onwards. In the Northern Hemisphere, the proportion remained consistently low from 2016 to 2018, with only 1 SARI case per 100 inpatients, followed by a significant increase in 2019–4 cases per 100 inpatients, which persisted in 2020–2023. Meanwhile, in the Southern Hemisphere, the proportion remained relatively stable from 2018 to 2022, with 1 or 2 SARI cases per 100 in-patients. Among the three ITZ, South Asia exhibited increased levels, with a peak of 96 cases per 1000 outpatients in 2020, while South East Asia maintained the lowest rates, peaking at 40 cases per 1000 outpatients in 2016. Fluctuations are evident across all zones, with East Asia experiencing a notable increase in 2019, South Asia displaying slight variations, and South East Asia showing a decline in 2021 followed by a slight rise in 2023. The fatality rates for SARI exhibited an upward trend from 2015 to 2017, followed by a dip in 2018 and beyond. The fatality rate was the highest in 2017 (29 per 1000 SARI cases) (Table 3) (Figs 4 and 5).

**Table 3:** Summary of ILI and SARI cases, 2015-2023 (Source: FluID).

Year	2015	2016	2017	2018	2019	2020	2021	2022	2023	Total
Total number of out-patient (OPD) cases*	124.37	56.53	129.76	121.43	130.92	16.80	2.50	3.83	9.13	**595.27**
Number of ILI cases*	4.54	2.14	4.30	3.83	4.18	0.67	0.08	0.22	0.40	**20.35**
Number of ILI cases per 1000 OPD cases	37	38	33	32	32	40	31	58	44	**34**
Total number of inpatients*	0.02	0.85	0.88	0.99	1.22	0.85	0.97	1.46	1.67	**8.89**
Number of SARI cases, i.e., only reported cases	3758	9676	11142	9718	43638	27727	44009	69455	64207	**283330**
Number of SARI cases per 100 inpatients	20.86	1.14	1.27	0.98	3.59	3.25	4.52	4.77	3.85	**3.19**
Number of SARI Deaths	8	76	321	98	163	147	65	85	104	**1067**
Fatality rate per 1000 SARI cases	2	8	29	10	4	5	1	1	2	**4**

*Approximate numbers in millions.

## Discussion

Influenza shows complex spatial and seasonal dynamics in SEAR [19]. Analysis of laboratory and epidemiological data reported to the RespiMART of GISRS (FluNet and FluID data) from SEAR MS (2015−2023) revealed region specific influenza transmission dynamics, virus subtypes, and regional patterns. Among SEAR member states, 14.2% of processed samples were laboratory-confirmed influenza, with India contributing the highest number of samples, given its population size. Peaks in influenza activity in SEAR was observed in 2017, 2019 and 2021 while notable declines occurred in 2020 and 2022. This is likely to be linked to the disruptions caused by COVID-19 pandemic [20]. WHO worked with MS to re-purpose, re-establish ILI/SARI surveillance and integrate Influenza and SARS-CoV-2 to sentinel surveillance with introduction of duplex testing for both pathogens. Subsequently reinstating surveillance to pre-COVID levels is evident in the 2021 and 2022 with upward data trends from member states [21].

The region showed distinct transmission patterns across MS located in Northern and Southern hemispheres and transmission zones (East Asia, Southeast Asia, and South Asia). While Influenza A, particularly A(H1N1)pdm 2009 subtype, remained dominant, Influenza B type demonstrated significant activity. Notably, B(Yamagata) lineage has shown a marked decline in circulation throughout SEAR, reflecting global trends. These variations in epidemiology suggest the need for zone-specific surveillance and vaccination strategies. Findings from the regression analysis further highlighted this heterogeneity, indicating that proportion of influenza-positive samples (FluNet) and the South Asia transmission zone were significant predictors of SARI cases across the region. Within this zone, Bhutan, Maldives, and Nepal demonstrated distinct associations after adjusting for seasonality, COVID-19 period, socioeconomic status and vaccine introduction of each country of SEAR. This suggests that observed differences likely also reflect variations in case reporting and surveillance coverage rather than variations in true disease burden only.

However, possible heterogeneity in the procedures and reporting patterns used by the laboratories, possible under/ skewed representativeness of the samples across seasons and geographies, selection of non-sentinel samples in case of unmet sample thresholds and inconsistent documentation and reporting practices make scenario planning and trend predictions complex. There is a need to strengthen reporting practices for FluID across SEAR MS with addition of more minimum essential variables as defined by the GISRS for standardised reporting and specification of denominators for calculation of proportions [22]. While WHO conducts External Quality Assurance Program that is mandatory for all NICs, in-country quality assurance programs could strengthen quality of diagnosis at sub-national laboratories that report to the central NIC in large countries where testing has been de-centralised with the NIC overseeing the quality. The same applies for in-country quality checks for data reported from sentinel surveillance sites.

A substantial proportion of samples for Influenza B was tagged as ‘lineage not determined’ across SEAR MS with some MS showing a very high proportion (e.g., Sri Lanka). WHO GISRS mandates that all such samples should be sent to one of the designated WHO Collaborating Centres for Influenza for typing, lineage determination and genomic sequencing. Improved characterization of influenza B lineages would be helpful in considering them for including in the influenza vaccine composition during vaccine composition meetings. Sharing of such strains with the GISRS is important for proper antigenic match of influenza vaccine viruses with those circulating for better efficacy of influenza vaccines as a preventive tool [23]. WHO and MS are encouraged to explore the underlying reasons affecting influenza surveillance including virus sharing with GISRS as a part of the agreed upon Terms of Reference of NICs. Both MS and WHO with the support of partners should undertake needful corrective measures which could include improving standards of influenza surveillance, close monitoring of timeliness and completeness of surveillance data reported, robust implementation of standard operating procedures of both surveilance and laboratory diagnosis, external and internal quality assurance procedures for laboratory diagnosis, genomic sequencing and allocation of resources for virus sharing for further characterisation, genetic, antigenic and serological testing. Also needed is building capacity of laboratories for Influenza B characterization or using referral services available at WHO collaborating centres, in particular. Since there are varying capacities and expertise in this regard in the region among MS, efforts are now underway for improvement of these areas with financial support, wider intra-regional advocacy, technical collaboration with WHO Collaborating Centres and H5 reference laboratories and cross-learning between NICs in the region.

Our study has several limitations. Data from the sentinel surveillance sites was inconsistent across months and years. Age and morbidity-stratified data was also inconsistent to analyse. Healthcare seeking behaviours of the people and reporting capacity of member states in SEAR are heterogeneous; we did not have information on the same and hence could not comment on its effects on the estimates we provided (S8 Table). WHO’s updated position paper on influenza vaccines (2022) has suggested that influenza vaccines should be administered within four months before the seasonal peak in countries with clear seasonal pattern and prior to the primary peak in countries with multiple peaks for maximal effectiveness [24]. WHO’s Strategic Advisory Group of Experts on Immunization (SAGE) also have recommended that timing of influenza vaccination should be based on the review of disaggregated national and sub-national epidemiological surveillance data. We, however, failed to infer on this since the SEAR MS showed mixed seasonality and at times, two peaks within the same year. Thus, national programmes will have to define the primary peak for national decision making on the timing of vaccination in their respective countries in consultation with the National Immunization Technical Advisory Group (NITAG) when seasonal influenza vaccination polices will be developed or updated.

The World Health Assembly’s (WHA) Executive Board Meeting held in January 2022 reiterated that sharing of Influenza Viruses of Pandemic Potential is critical for effective pandemic influenza preparedness including risk assessment, preparation of appropriate Medical Counter Measures and timely response with equity [25]. The 13^th^ General Programme for Work(GPW) (2019−23) of WHO that targets to better protect 1 billion more people against health emergencies and the Five-Year Regional Strategic Plan to Strengthen Public Health Preparedness and Response (2019–23) emphasized upon influenza preparedness in alignment with Global Influenza Strategy (2019−30) [26,27]. GPW 14 continues the same agenda uninterruptedly. Effective implementation of WHO’s influenza preparedness agenda requires addressing regional disparities in surveillance capacity while focusing primarily on further strengthening sentinel surveillance networks at the same time implementing mosaic surveillance approach to supplement evidence generation in areas where sentinel surveillance is deemed insufficient across member states. This aligns with the Global Influenza Strategy (2019−30), Pandemic Influenza Preparedness (PIP) Framework, PIP framework’s High Level Implementation Plan III (2024−30), its PIP Partnership Contributions (PIP-PC) for preparedness and response and the respiratory diseases module of the Preparedness and Resilience for Emerging Threats (PRET) initiative.

## Conclusion

Influenza epidemiology shows spatial and seasonal variability in SEAR. FluNet and FluID data sharing platforms of the GISRS serve as efficient systems for observing achievement of primary and secondary surveillance objectives set in WHO guidelines for MS such as determining influenza trends, seasonality, circulating strains etc. However, the performance of these surveillance systems in relation to GISRS standards is heterogenous across SEAR MS. In general, improved reporting of epidemiological data, reaching WHO minimal threshold of samples processed for testing and better linkages between epidemiological and laboratory data are areas for further focus in SEAR MS. Empirically reflecting this need, we noted that the FluNet and FluID datasets needed better linking and reconciliation as the correlation between the two variables was weak in our analysis.

Going forward, there is a need to address regional disparity in achieving WHO recommended influenza surveillance standards and further strengthen the functionality of surveillance networks to achieve these minimum standards in SEAR MS through collaborative efforts among member states with support from WHO and other partners such as WHO Collaborating Centres. By sharing best practices, resources, and expertise, countries can collectively strengthen their ability to detect and respond to influenza threats. As SEARO MS share borders with population movements, countries mutually benefit from such cross- border collaborations. This will be boosted by the WHO’s recommendations for expanded GISRS where influenza sentinel surveillance network is leveraged for integrated surveillance of pan respiratory pathogens of epidemic and pandemic potential. The right sizing of sentinel sites for better quality of data generated against focussing on a large number of sentinel surveillance sites compromising quality for merely ensuring geographic representativeness of sentinel sites is a key area for the national programmes to work on with WHO and partners. Key priorities include optimizing numbers of sentinel sites, achieving minimum essential number of samples processed for testing, ensuring coverage and quality of data, while leveraging existing influenza networks for integrated surveillance of respiratory pathogens with epidemic and pandemic potential. This expanded GISRS surveillance network is built on lessons learned from influenza surveillance and COVID-19 response, aiming to create more robust, data-driven early warning, alert and response system for preparedness for future epidemics/ pandemics as well as utilizing the same system for monitoring response during the epidemic/ pandemic.

## Supporting information

S1 File Dataset for FluNet. (XLSX)

S2 File Dataset for FluID. (XLSX)

S3 Fig Trend of influenza cases in WHO SEAR Member States, 2015-2023 (PDF)

S4 Fig Circulation of Influenza virus according to Hemisphere. (PDF)

S5 Fig Circulation of Influenza virus according to Influenza Transmission Zones. (PDF)

S6 Fig Proportion of influenza A and B by subtypes in WHO SEAR member states from 2015–2023. (PDF)

S7 Fig Number and the proportion of Influenza-like Illness (ILI) cases per 1000 outpatients and Severe Acute Respiratory Infection (SARI) cases per 100 inpatient, SEAR (2015–2023). (PDF)

S8 Table Data gaps observed according to the sequence of analysis. (PDF)

## References

[1] Western Pacific Region Global Influenza Surveillance and Response System. Epidemiological and virological characteristics of influenza in the Western Pacific Region of the World Health Organization, 2006-2010. PLoS One. 2012;7(5):e37568. doi: 10.1371/journal.pone.0037568 22675427 PMC3366627

[2] Influenza (Seasonal). [cited 15 Apr 2024]. Available: https://www.who.int/news-room/fact-sheets/detail/influenza-(seasonal)

[3] WHO Mosaic Respiratory Surveillance Framework. [cited 28 Jun 2025]. Available: https://www.who.int/initiatives/mosaic-respiratory-surveillance-framework

[4] Strengthening response to pandemics and other public-health emergencies. [cited 13 Jun 2025]. Available: https://www.who.int/publications/i/item/strengthening-response-to-pandemics-and-other-public-health-emergencies

[5] Fineberg HV. Pandemic preparedness and response--lessons from the H1N1 influenza of 2009. N Engl J Med. 2014;370(14):1335–42. doi: 10.1056/NEJMra1208802 24693893

[6] Report of the Review Committee on the Functioning of the International Health Regulations (2005) in relation to Pandemic (H1N1) 2009. World Health Organization; 2011. Available: https://apps.who.int/gb/ebwha/pdf_files/WHA64/A64_10-en.pdf

[7] Flunet. [cited 28 Sep 2022]. Available: https://www.who.int/tools/flunet

[8] National Influenza Centres. [cited 28 Sep 2022]. Available: https://www.who.int/initiatives/global-influenza-surveillance-and-response-system/national-influenza-centres

[9] About WHO in the South-East Asia Region. [cited 22 Apr 2024]. Available: https://www.who.int/southeastasia/about

[10] Nii-Trebi NI. Emerging and Neglected Infectious Diseases: Insights, Advances, and Challenges. Biomed Res Int. 2017;2017:5245021. doi: 10.1155/2017/5245021 28286767 PMC5327784

[11] PIP partnership contribution fund recipient countries in WHO’s Southeast Asia region meet in New Delhi to plan for Pandemic Influenza Preparedness in the 2024-25 biennium. [cited 15 Nov 2024]. Available: https://www.who.int/southeastasia/news/detail/15-10-2023-pip-pret-rm

[12] Pandemic influenza preparedness framework: partnership contribution high-level implementation plan III 2024-2030. [cited 15 Nov 2024]. Available: https://www.who.int/publications/i/item/9789240070141

[13] Members of the WHO South-East Asia Region Global Influenza Surveillance and Response System. Seasonal influenza surveillance (2009-2017) for pandemic preparedness in the WHO South-East Asia Region. WHO South East Asia J Public Health. 2020;9(1):55–65. doi: 10.4103/2224-3151.282999 32341224

[14] Maintaining essential health services: operational guidance for the COVID-19 context: interim guidance, 1 June 2020. [cited 21 Jun 2025]. Available: https://www.who.int/publications/i/item/WHO-2019-nCoV-essential_health_services-2020.2

[15] Asia WHORO for S-E. Implementation of WHO guidance on maintaining influenza surveillance and monitoring of SARS-CoV-2 through national surveillance systems during the COVID-19 pandemic in the SEA Region Member States. 2021 [cited 21 Jun 2025]. Available: https://iris.who.int/handle/10665/350448

[16] Staadegaard L, Del Riccio M, Wiegersma S, El Guerche-Séblain C, Dueger E, Akçay M, et al. The impact of the SARS-CoV-2 pandemic on global influenza surveillance: Insights from 18 National Influenza Centers based on a survey conducted between November 2021 and March 2022. Influenza Other Respir Viruses. 2023;17(5):e13140. doi: 10.1111/irv.13140 37180840 PMC10173050

[17] Influenza surveillance outputs. [cited 15 Apr 2024]. Available: https://www.who.int/teams/global-influenza-programme/surveillance-and-monitoring/influenza-surveillance-outputs

[18] Surveillance case definitions for ILI and SARI. [cited 13 Jun 2025]. Available: https://www.who.int/teams/global-influenza-programme/surveillance-and-monitoring/case-definitions-for-ili-and-sari

[19] Coletti P, Poletto C, Turbelin C, Blanchon T, Colizza V. Shifting patterns of seasonal influenza epidemics. Sci Rep. 2018;8(1):12786. doi: 10.1038/s41598-018-30949-x 30143689 PMC6109160

[20] Regional meeting on implementation of WHO guidance on maintaining influenza surveillance and monitoring SARSCoV-2 through national sentinel surveillance systems during the COVID-19 pandemic in SEAR Member States. [cited 28 Sep 2022]. Available: https://www.who.int/southeastasia/news/events/detail/2021/01/13/south-east-asia-events/regional-meeting-on-implementation-of-who-guidance-on-maintaining-influenza-surveillance-and-monitoring-sarscov-2-through-national-sentinel-surveillance-systems-during-the-covid-19-pandemic-in-sear-member-states

[21] South-East Asia: Influenza virus sharing in a time of COVID-19. [cited 28 Sep 2022]. Available: https://www.who.int/news/item/03-12-2021-south-east-asia-influenza-virus-sharing-in-a-time-of-covid-19

[22] Seventy years of GISRS – the Global Influenza Surveillance & Response System. [cited 28 Sep 2022]. Available: https://www.who.int/news-room/feature-stories/detail/seventy-years-of-gisrs---the-global-influenza-surveillance---response-system

[23] El Guerche-Séblain C, Caini S, Paget J, Vanhems P, Schellevis F. Epidemiology and timing of seasonal influenza epidemics in the Asia-Pacific region, 2010-2017: implications for influenza vaccination programs. BMC Public Health. 2019;19(1):331. doi: 10.1186/s12889-019-6647-y 30898100 PMC6429768

[24] Feng LZ, Jiang HY, Yi J, Qian LL, Xu JD, Zheng LB, et al. Introduction and implications of WHO position paper: vaccines against influenza, May 2022. Zhonghua Yi Xue Za Zhi. 2022;102(30):2315–8. doi: 10.3760/cma.j.cn112137-20220518-01090 35970790

[25] WHO Director-General’s opening remarks at the 150th session of the Executive Board — 24 January 2022. [cited 28 Sep 2022]. Available: https://www.who.int/director-general/speeches/detail/who-director-general-s-opening-remarks-at-the-150th-session-of-the-executive-board-24-january-2022

[26] Five-year Regional Strategic Plan to Strengthen Public Health Preparedness and Response. World Health Organization; 2019. Available: https://cdn.who.int/media/docs/default-source/searo/whe/five-year-regional-strategic-plan-to-strengthen-public-health-preparedness-and-response-(-2019-2023).pdf?sfvrsn=1ce7bb25_0

[27] Thirteenth General Programme Of Work 2019–2023. World Health Organization; 2019. Available: https://apps.who.int/iris/bitstream/handle/10665/324775/WHO-PRP-18.1-eng.pdf

