# Supplementary material for: An analysis of WHO FluNet and FluID influenza surveillance data for South East Asia Region, 2015–2023
Source: PLoS One. 2026 Feb 20;21(2):e0341567. doi: 10.1371/journal.pone.0341567 (PMC12923055; doi:10.1371/journal.pone.0341567)
Supplement: S3 Fig — (PDF) [file pone.0341567.s003.pdf]

S3: Trend of influenza cases in WHO SEAR Member States, 2015-2023

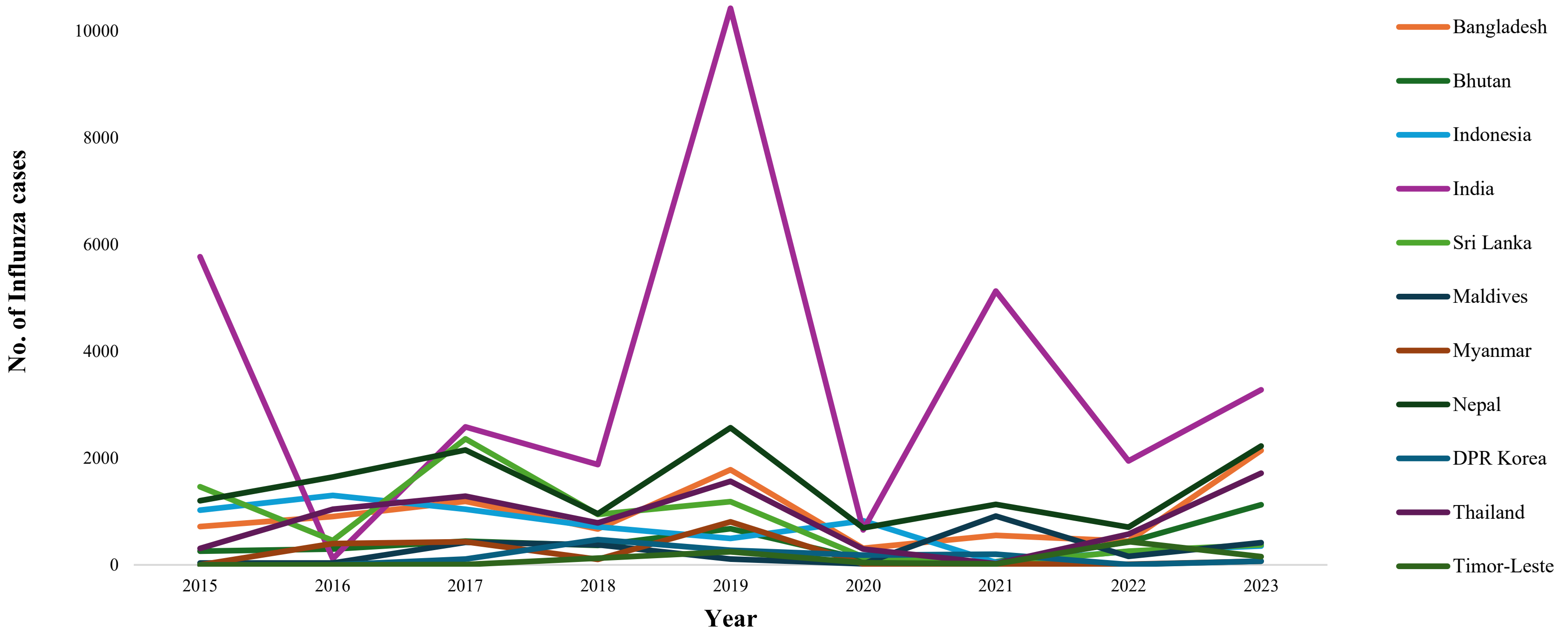

Figure S3.1: Trend of influenza cases in WHO SEAR Member States, 2015-2023

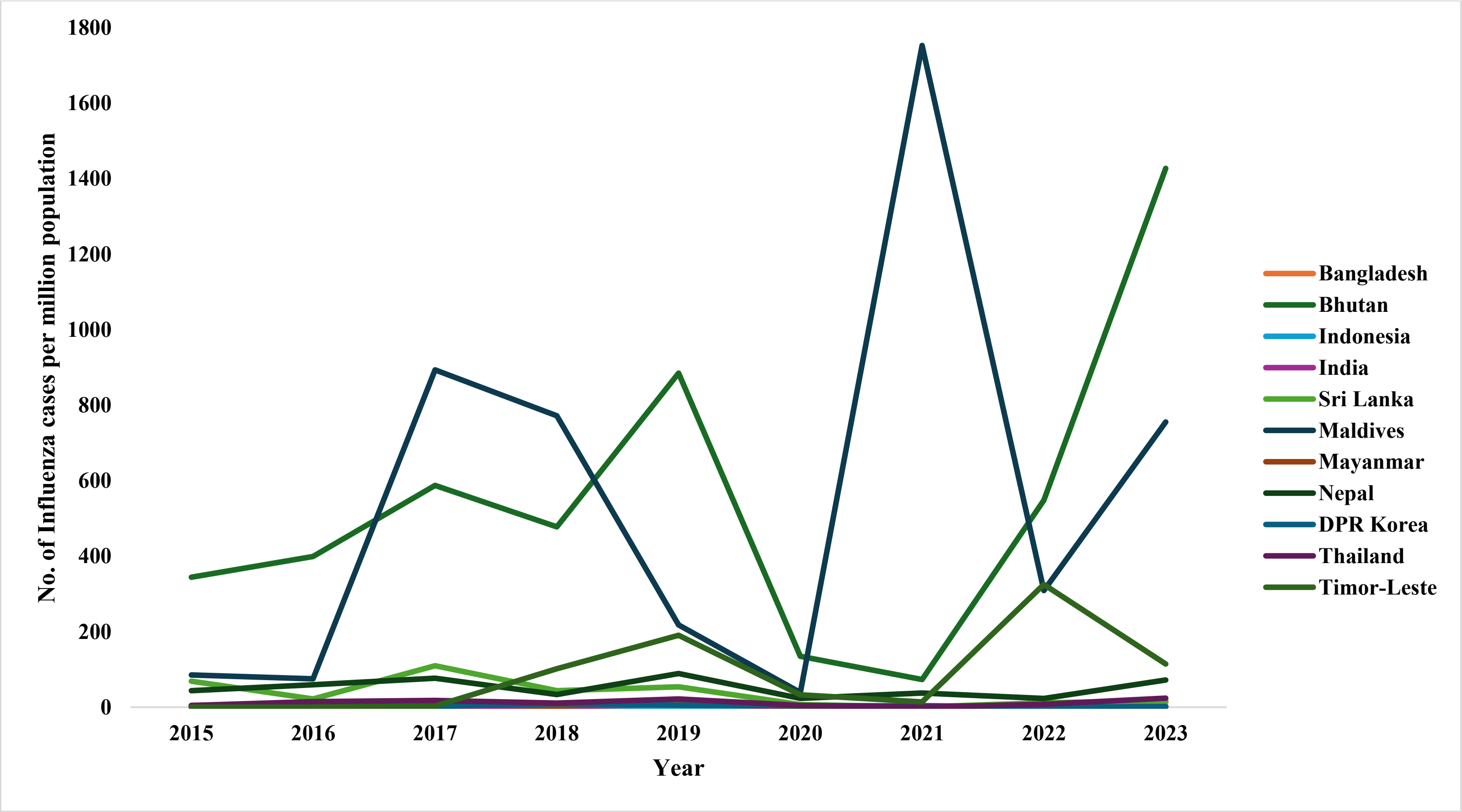

Data Source: World Bank (Mid-year population); FluNet (Influenza cases)

Figure S3.2: Influenza Cases per Million mid-year Population in SEAR, 2015-2023

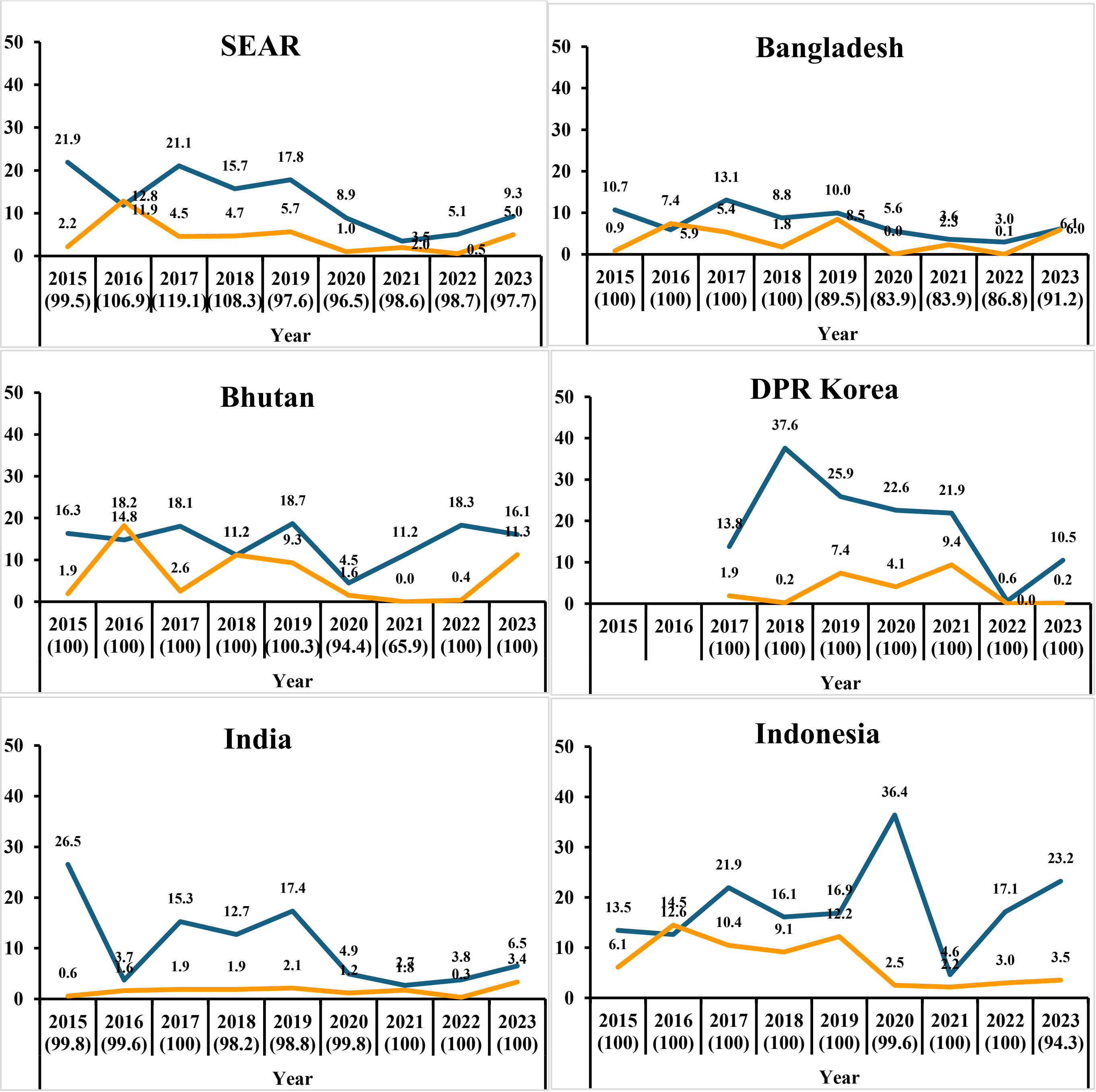

SEAR
2015 (99.5)
2016 (106.9)
2017 (119.1)
2018 (108.3)
2019 (97.6)
2020 (96.5)
2021 (98.6)
2022 (98.7)
2023 (97.7)
Year
Bangladesh
2015 (100)
2016 (100)
2017 (100)
2018 (100)
2019 (89.5)
2020 (83.9)
2021 (83.9)
2022 (86.8)
2023 (91.2)
Year
Bhutan
2015 (100)
2016 (100)
2017 (100)
2018 (100)
2019 (100.3)
2020 (94.4)
2021 (65.9)
2022 (100)
2023 (100)
Year
DPR Korea
2015
2016
2017 (100)
2018 (100)
2019 (100)
2020 (100)
2021 (100)
2022 (100)
2023 (100)
Year
India
2015 (99.8)
2016 (99.6)
2017 (100)
2018 (98.2)
2019 (98.8)
2020 (99.8)
2021 (100)
2022 (100)
2023 (100)
Year
Indonesia
2015 (100)
2016 (100)
2017 (100)
2018 (100)
2019 (100)
2020 (99.6)
2021 (100)
2022 (100)
2023 (94.3)
Year

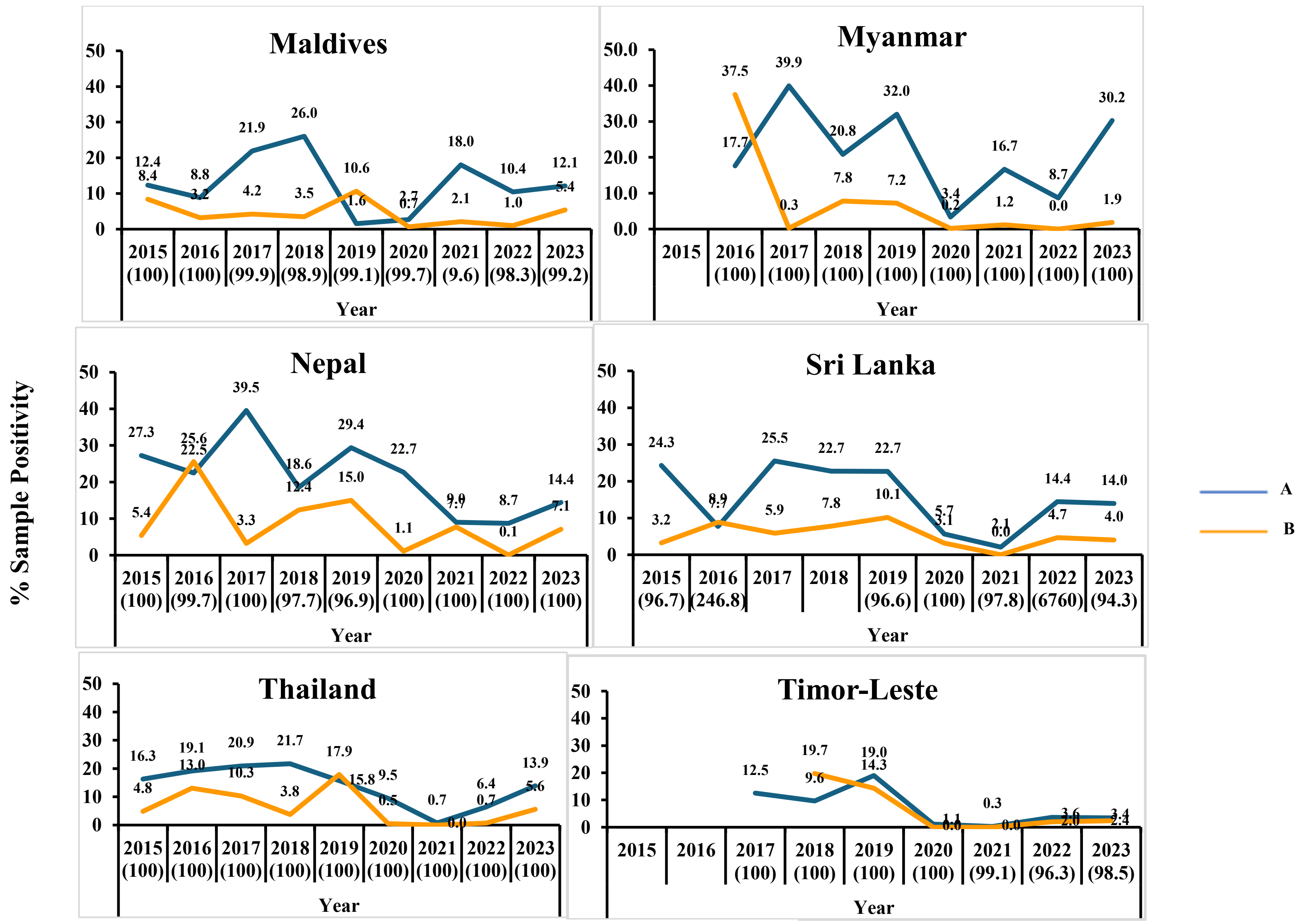

% of sample processed for each year is given in the () on the x-axis

Figure S3.3: Trend of Influenza A and B positivity rates in the WHO SEAR member states, 2015-2023
