# Supplementary material for: An analysis of WHO FluNet and FluID influenza surveillance data for South East Asia Region, 2015–2023
Source: PLoS One. 2026 Feb 20;21(2):e0341567. doi: 10.1371/journal.pone.0341567 (PMC12923055; doi:10.1371/journal.pone.0341567)
Supplement: S4 Fig — (PDF) [file pone.0341567.s004.pdf]

S4: Circulation of Influenza virus according to Hemisphere

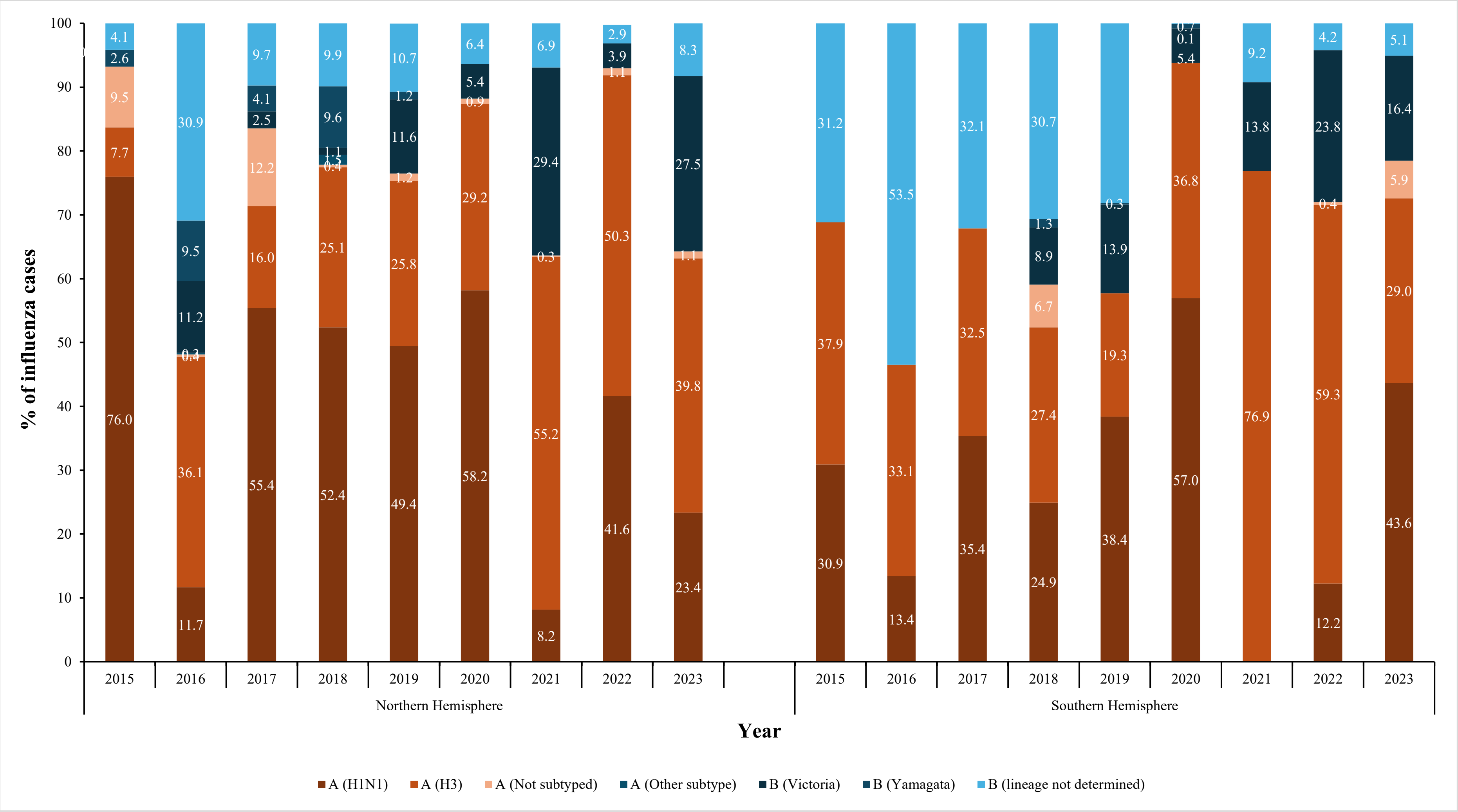

Figure S4.1: Circulation of Influenza A & B subtypes in Northern and Southern Hemisphere

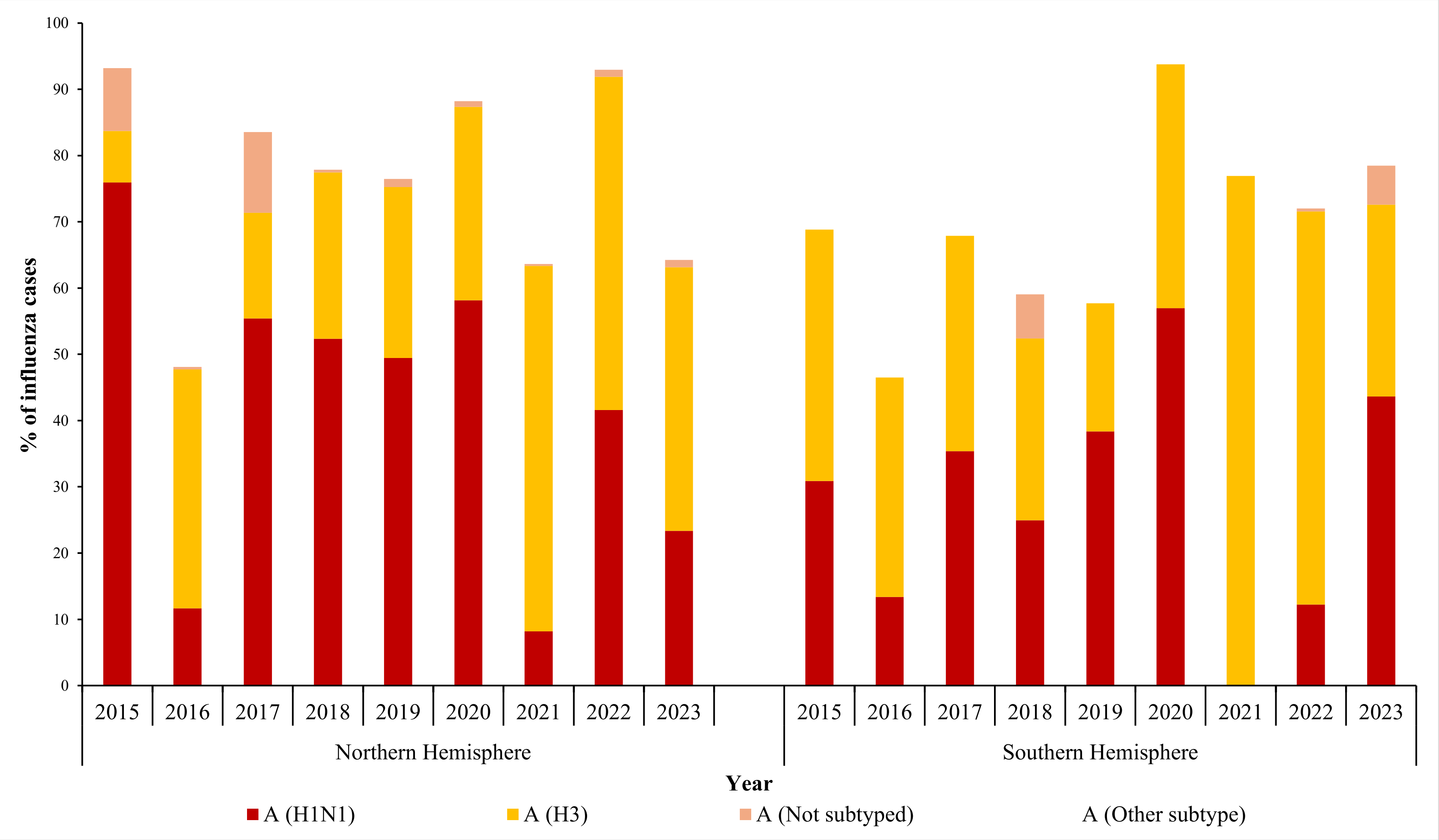

Figure S4.2: Circulation of Influenza A subtypes in Northern and Southern Hemisphere

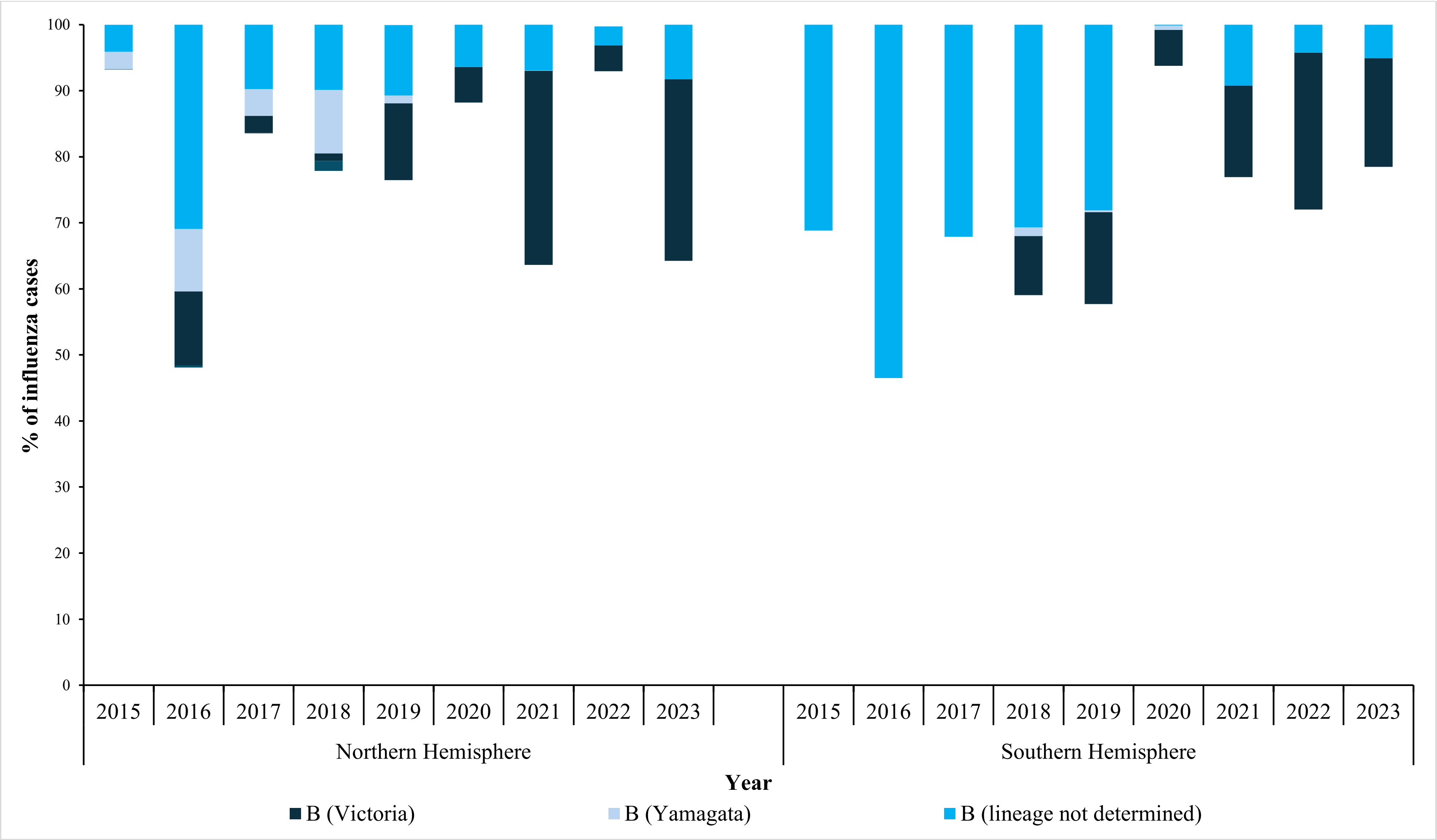

Figure S4.3: Circulation of Influenza B subtypes in Northern and Southern Hemisphere
