# Supplementary material for: An analysis of WHO FluNet and FluID influenza surveillance data for South East Asia Region, 2015–2023
Source: PLoS One. 2026 Feb 20;21(2):e0341567. doi: 10.1371/journal.pone.0341567 (PMC12923055; doi:10.1371/journal.pone.0341567)
Supplement: S5 Fig — (PDF) [file pone.0341567.s005.pdf]

S5: Circulation of Influenza virus according to Influenza Transmission Zones

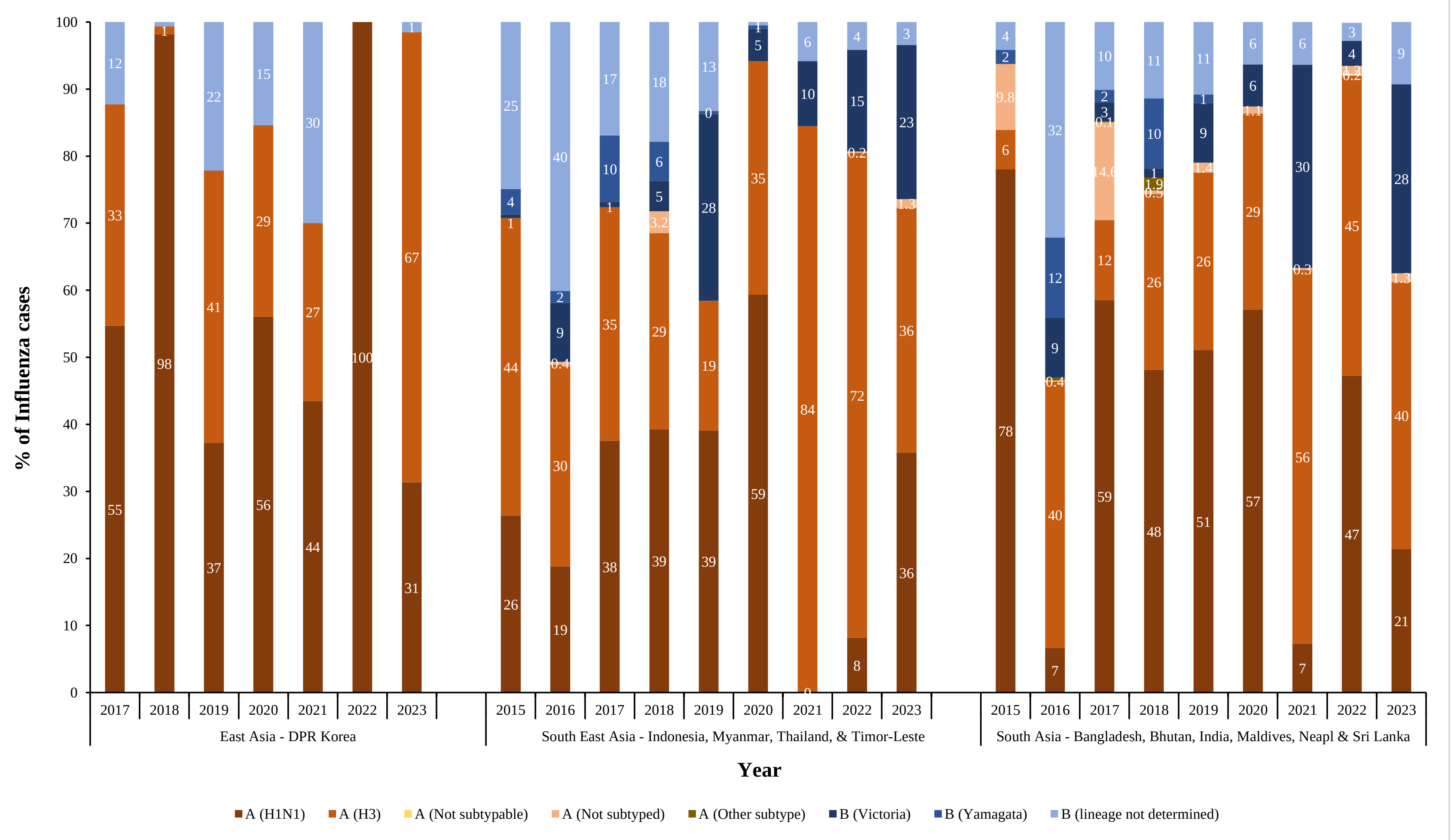

Fig. S5: Circulation of Influenza A and B subtypes in the Influenza Transmission Zones of SEAR, 2015-2023
