# Supplementary material for: An analysis of WHO FluNet and FluID influenza surveillance data for South East Asia Region, 2015–2023
Source: PLoS One. 2026 Feb 20;21(2):e0341567. doi: 10.1371/journal.pone.0341567 (PMC12923055; doi:10.1371/journal.pone.0341567)
Supplement: S6 Fig — (PDF) [file pone.0341567.s006.pdf]

S6: Proportion of influenza A and B by subtypes in WHO SEAR member states from 2015-2023

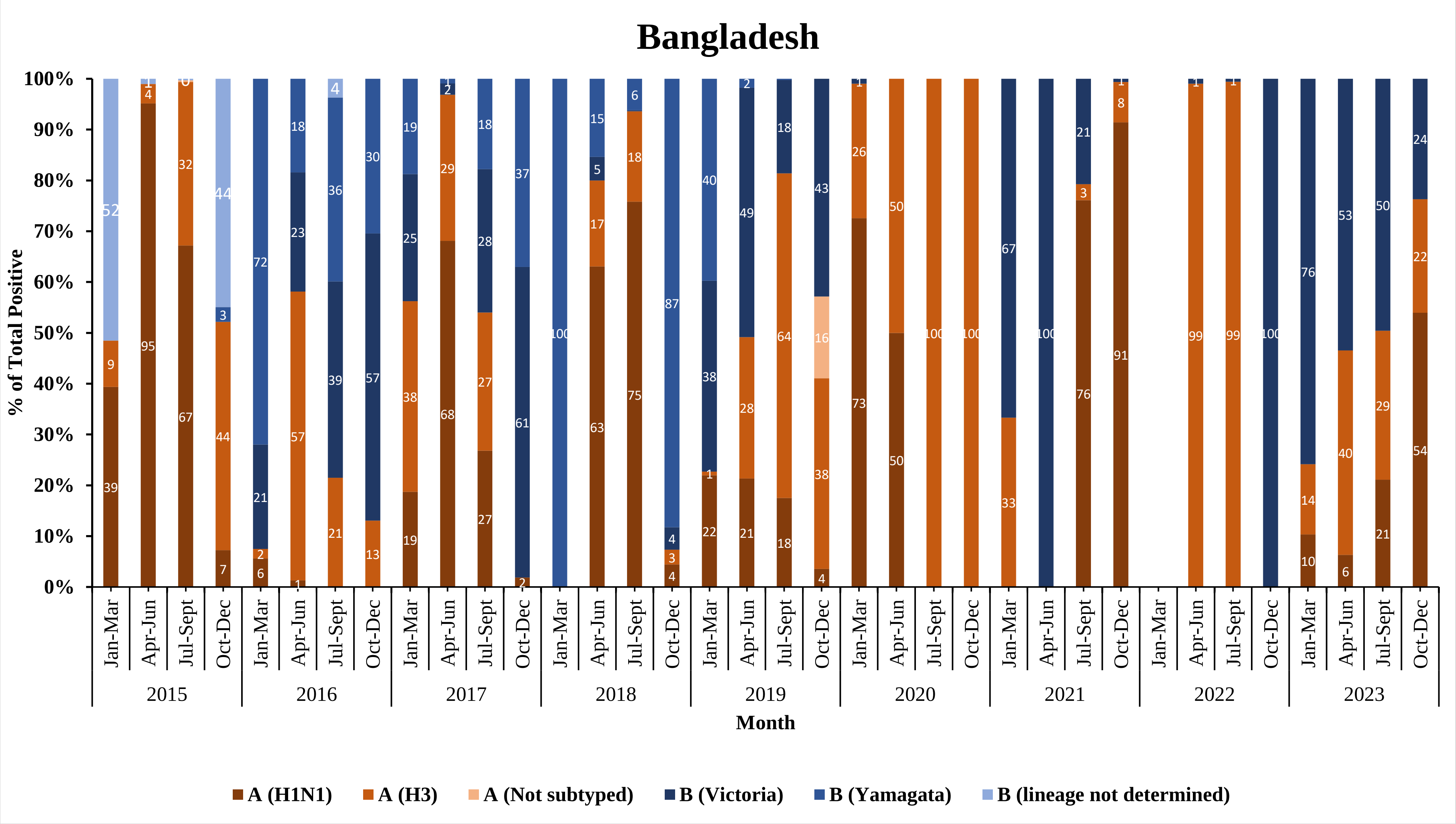

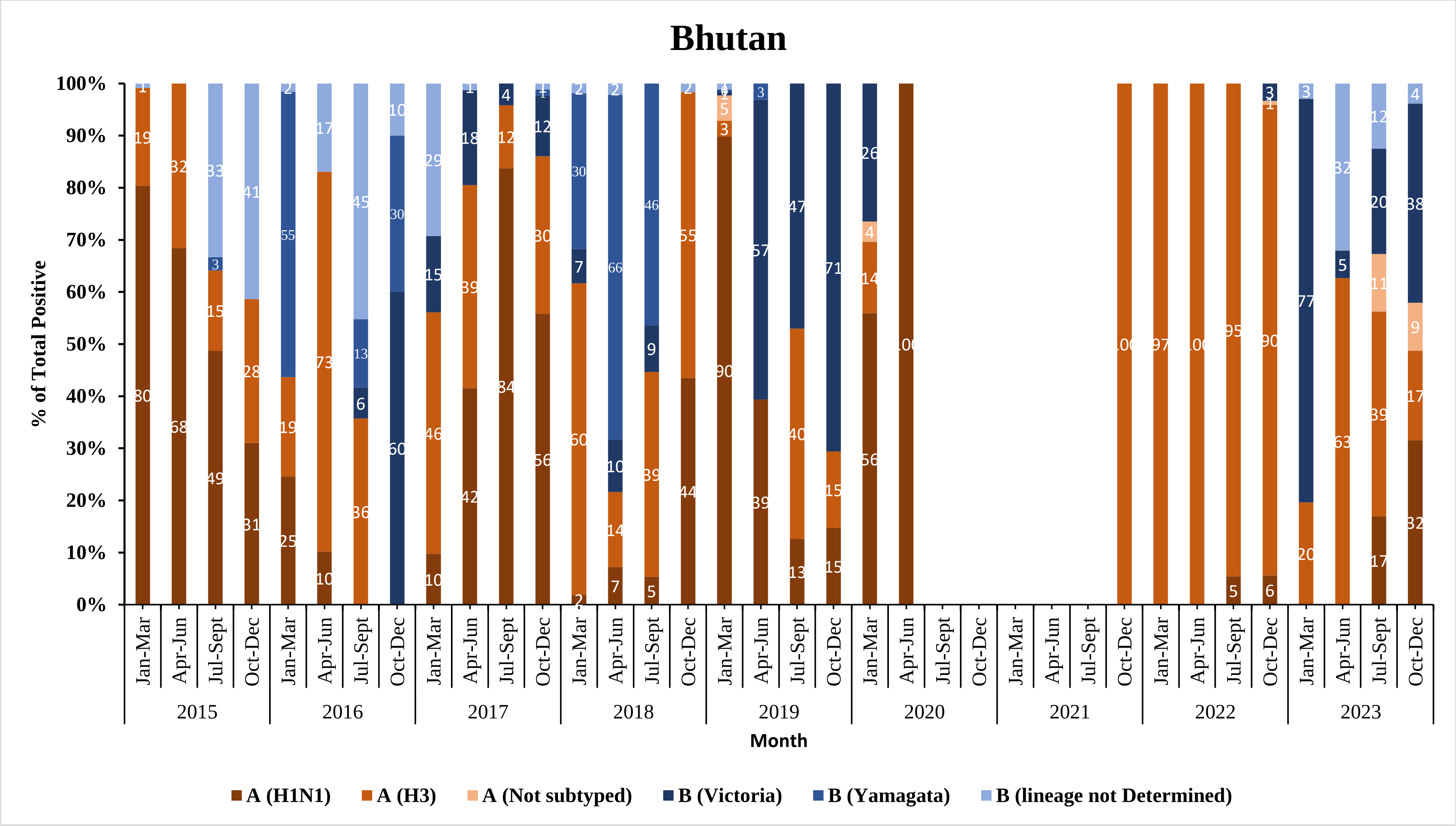

Bhutan
% of Total Positive
Month
2015
2016
2017
2018
2019
2020
2021
2022
2023
Jan-Mar
Apr-Jun
Jul-Sept
Oct-Dec
A (H1N1)
A (H3)
A (Not subtyped)
B (Victoria)
B (Yamagata)
B (lineage not Determined)

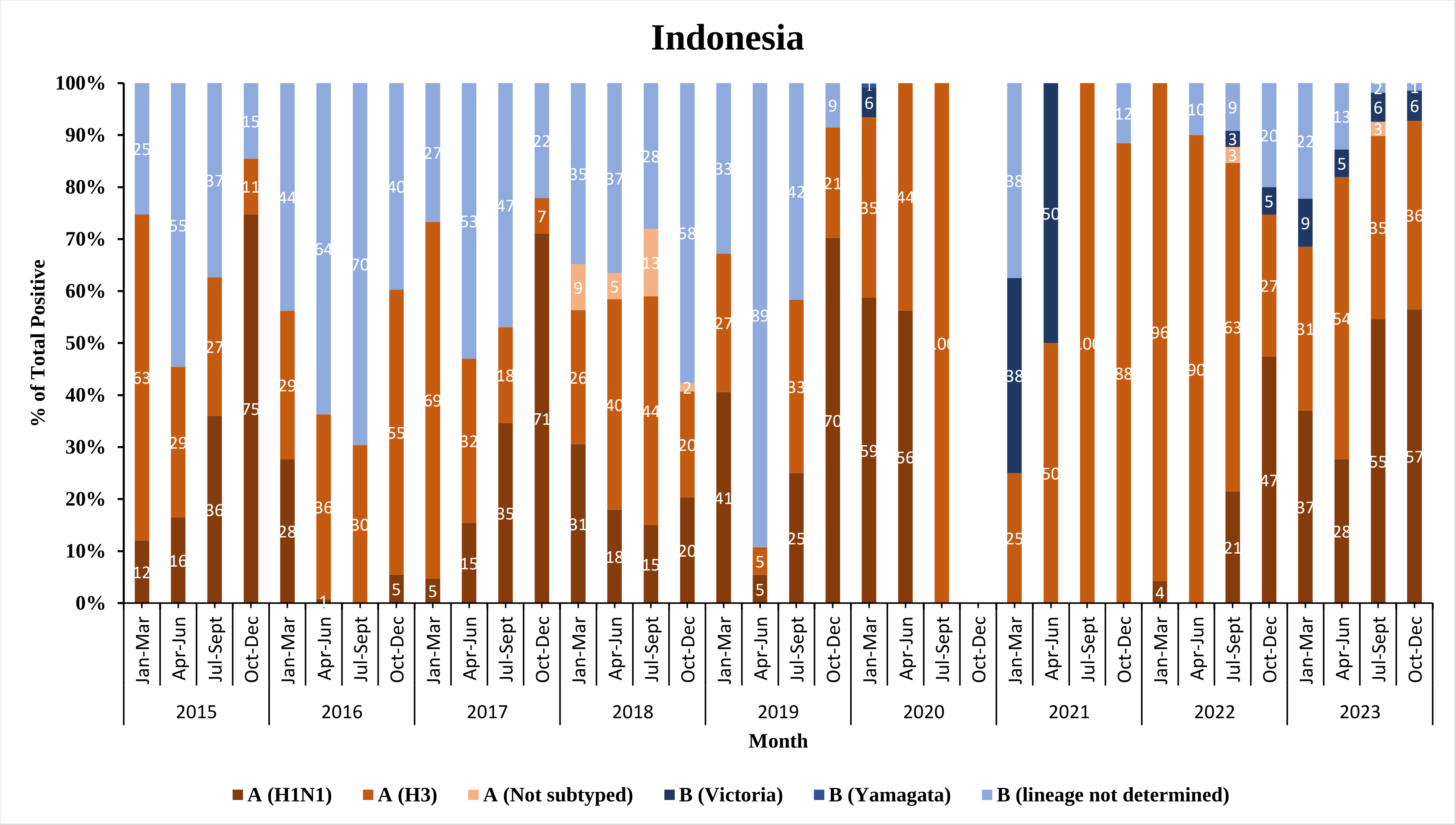

Indonesia
% of Total Positive
Month
2015
2016
2017
2018
2019
2020
2021
2022
2023
Jan-Mar
Apr-Jun
Jul-Sept
Oct-Dec
A (H1N1)
A (H3)
A (Not subtyped)
B (Victoria)
B (Yamagata)
B (lineage not determined)

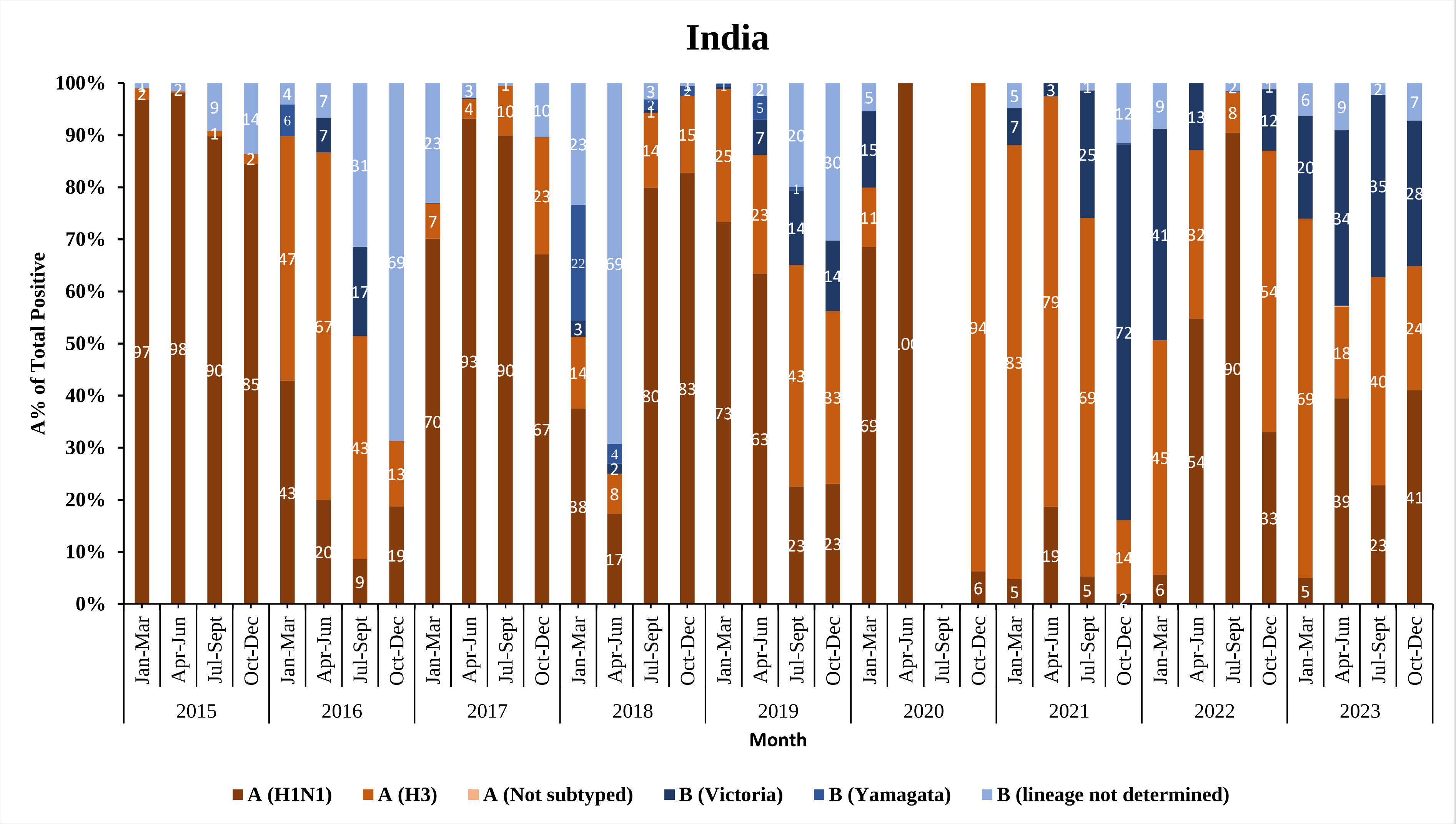
India
A% of Total Positive
Month
2015
2016
2017
2018
2019
2020
2021
2022
2023
Jan-Mar
Apr-Jun
Jul-Sept
Oct-Dec
A (H1N1)
A (H3)
A (Not subtyped)
B (Victoria)
B (Yamagata)
B (lineage not determined)

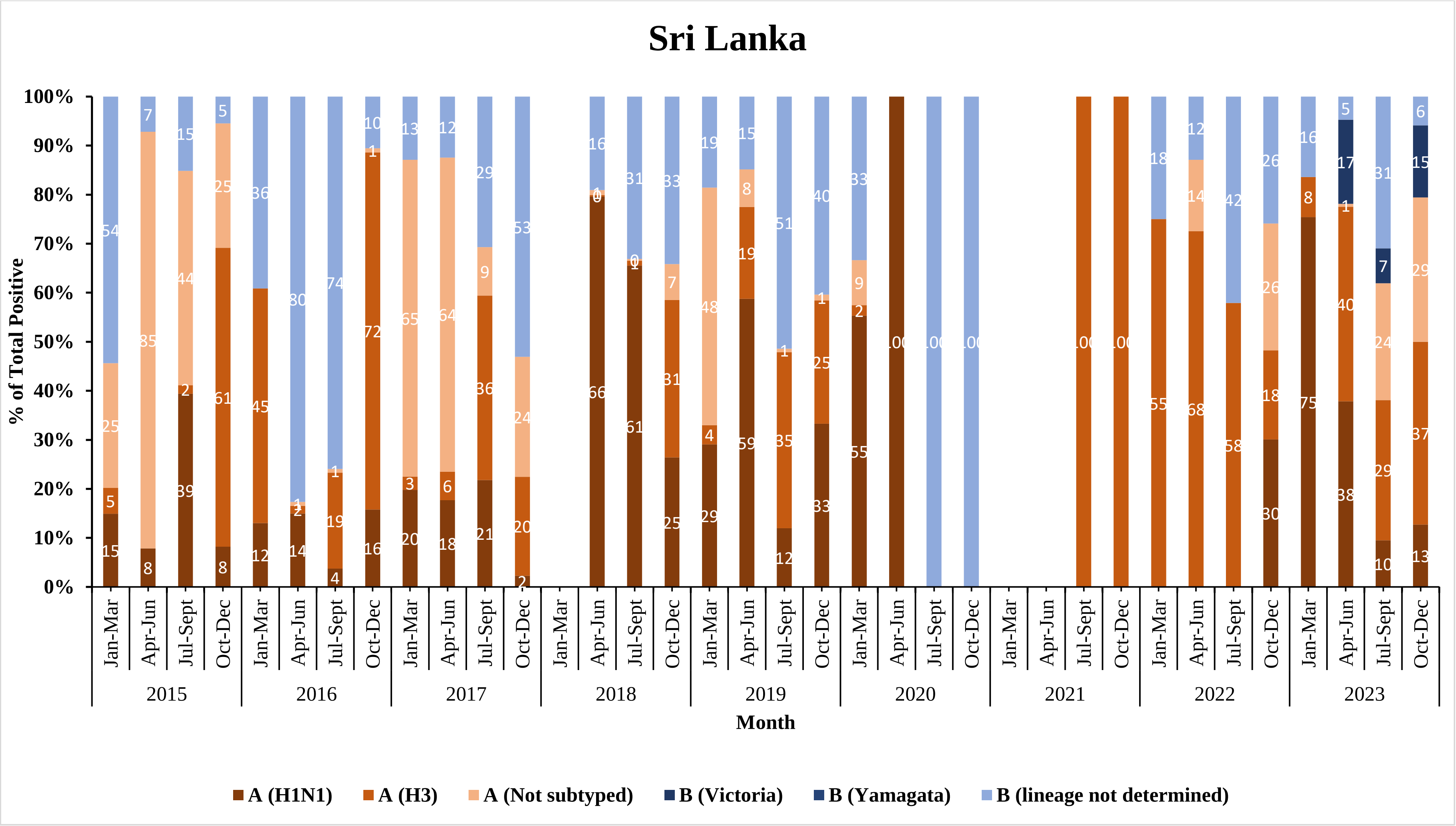
Sri Lanka
% of Total Positive
Month
2015
2016
2017
2018
2019
2020
2021
2022
2023
Jan-Mar
Apr-Jun
Jul-Sept
Oct-Dec
A (H1N1)
A (H3)
A (Not subtyped)
B (Victoria)
B (Yamagata)
B (lineage not determined)

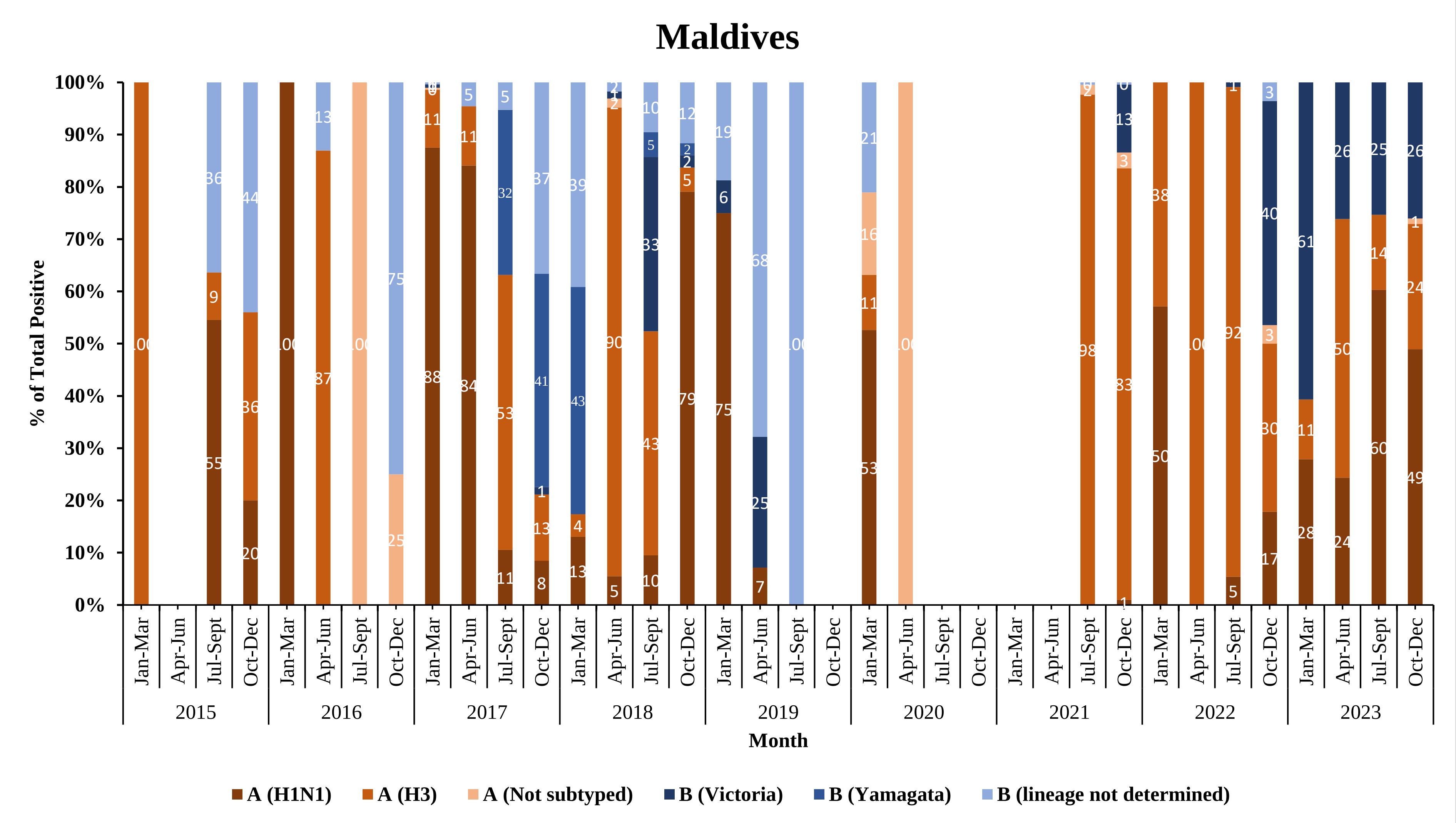
Maldives
% of Total Positive
100%
90%
80%
70%
60%
50%
40%
30%
20%
10%
0%
Jan-Mar
Apr-Jun
Jul-Sept
Oct-Dec
2015
2016
2017
2018
2019
2020
2021
2022
2023
Month
A (H1N1)
A (H3)
A (Not subtyped)
B (Victoria)
B (Yamagata)
B (lineage not determined)

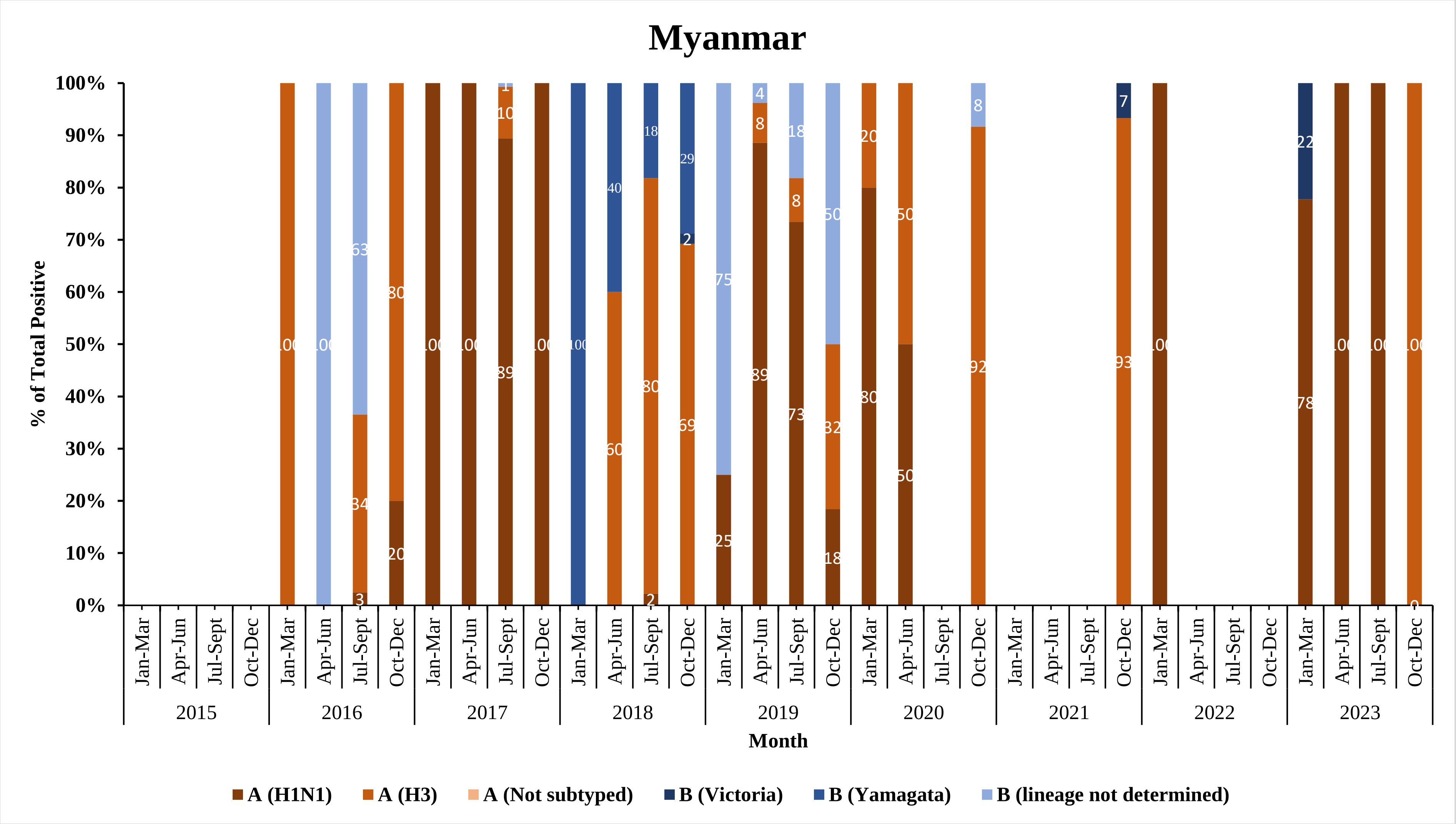

Myanmar
% of Total Positive
100%
90%
80%
70%
60%
50%
40%
30%
20%
10%
0%
Jan-Mar
Apr-Jun
Jul-Sept
Oct-Dec
2015
2016
2017
2018
2019
2020
2021
2022
2023
Month
A (H1N1)
A (H3)
A (Not subtyped)
B (Victoria)
B (Yamagata)
B (lineage not determined)

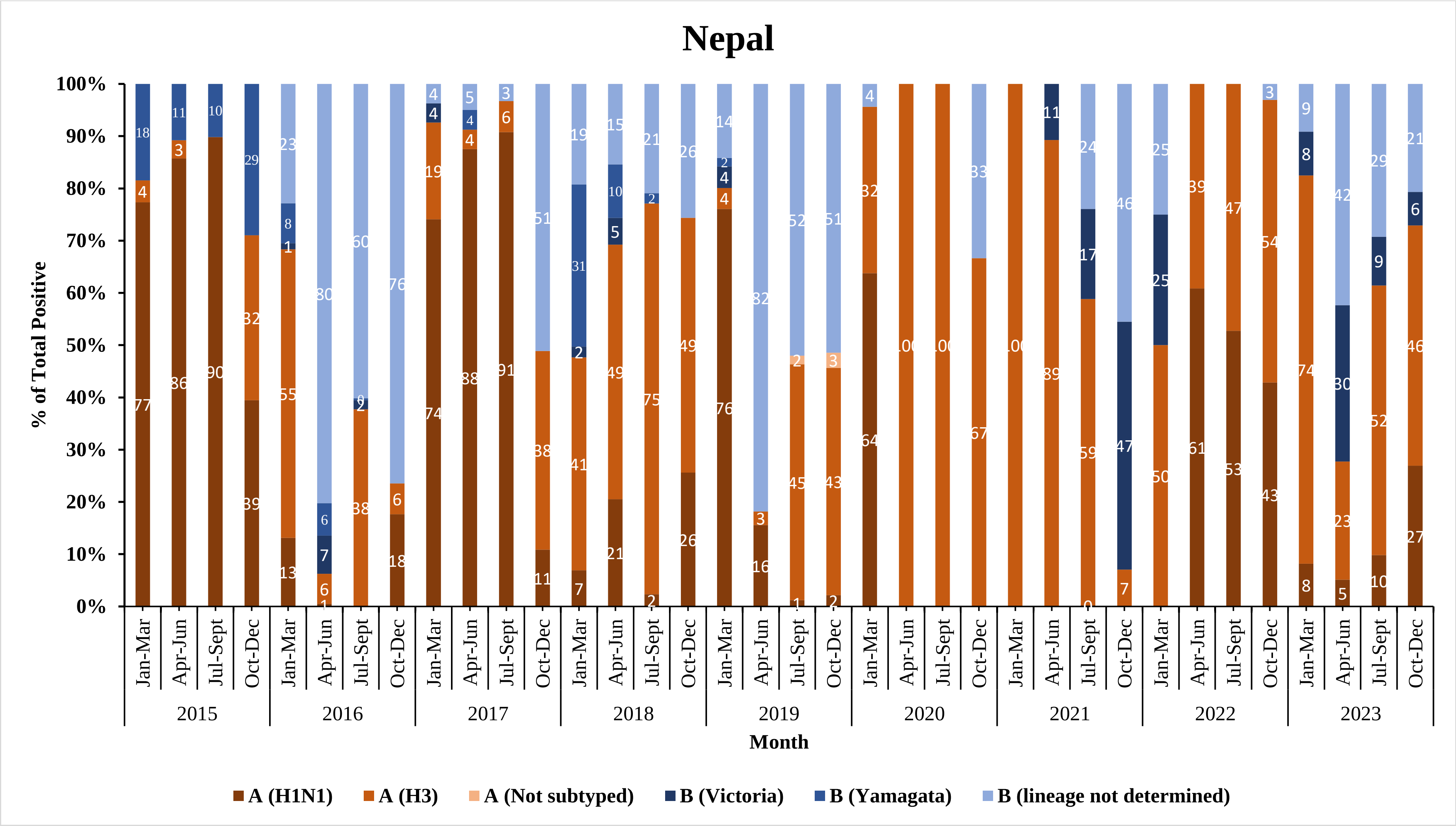
Nepal
100%
90%
80%
70%
60%
50%
40%
30%
20%
10%
0%
% of Total Positive
Jan-Mar
Apr-Jun
Jul-Sept
Oct-Dec
2015
2016
2017
2018
2019
2020
2021
2022
2023
Month
A (H1N1)
A (H3)
A (Not subtyped)
B (Victoria)
B (Yamagata)
B (lineage not determined)

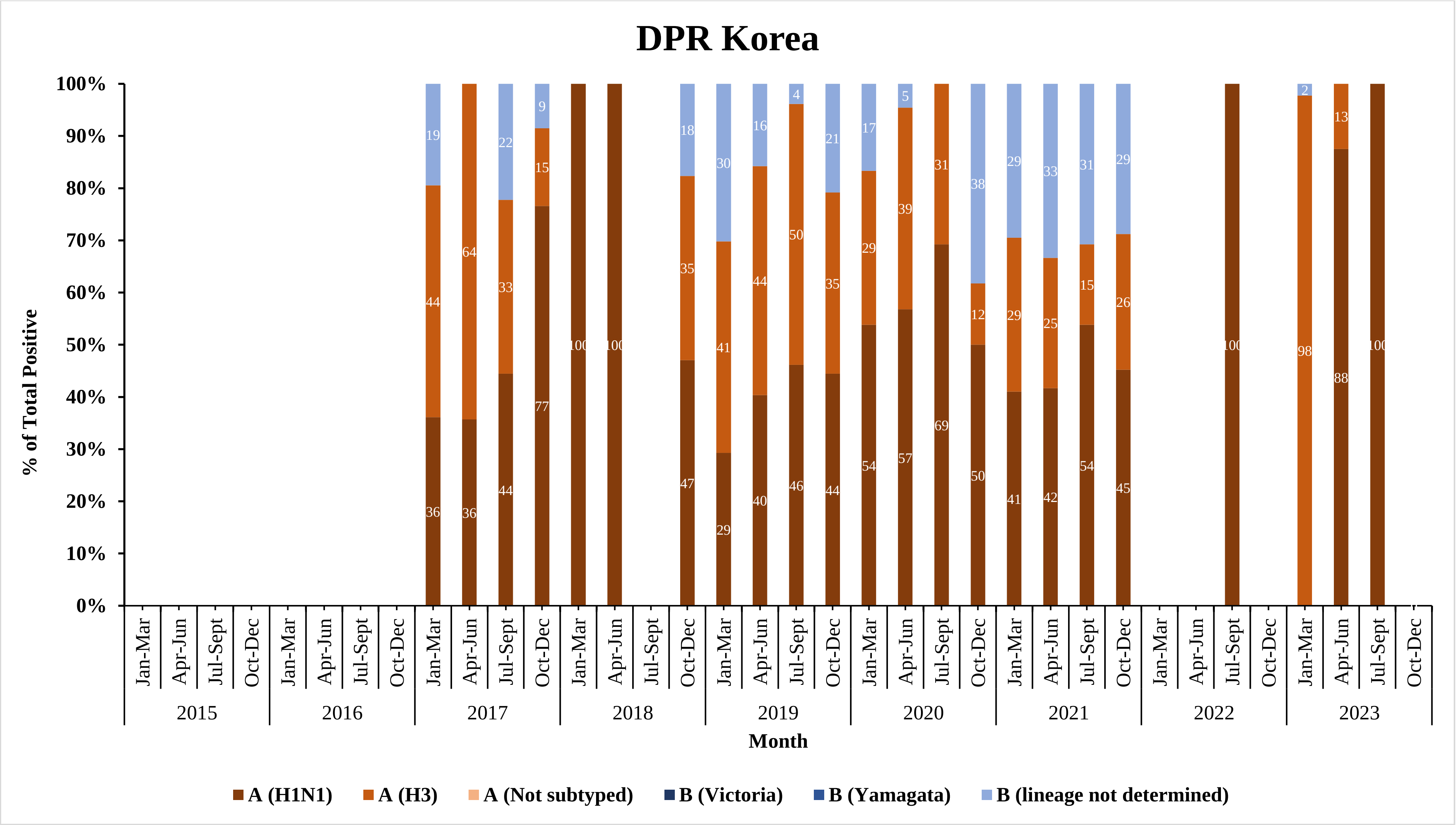
DPR Korea
% of Total Positive
Month
A (H1N1)
A (H3)
A (Not subtyped)
B (Victoria)
B (Yamagata)
B (lineage not determined)

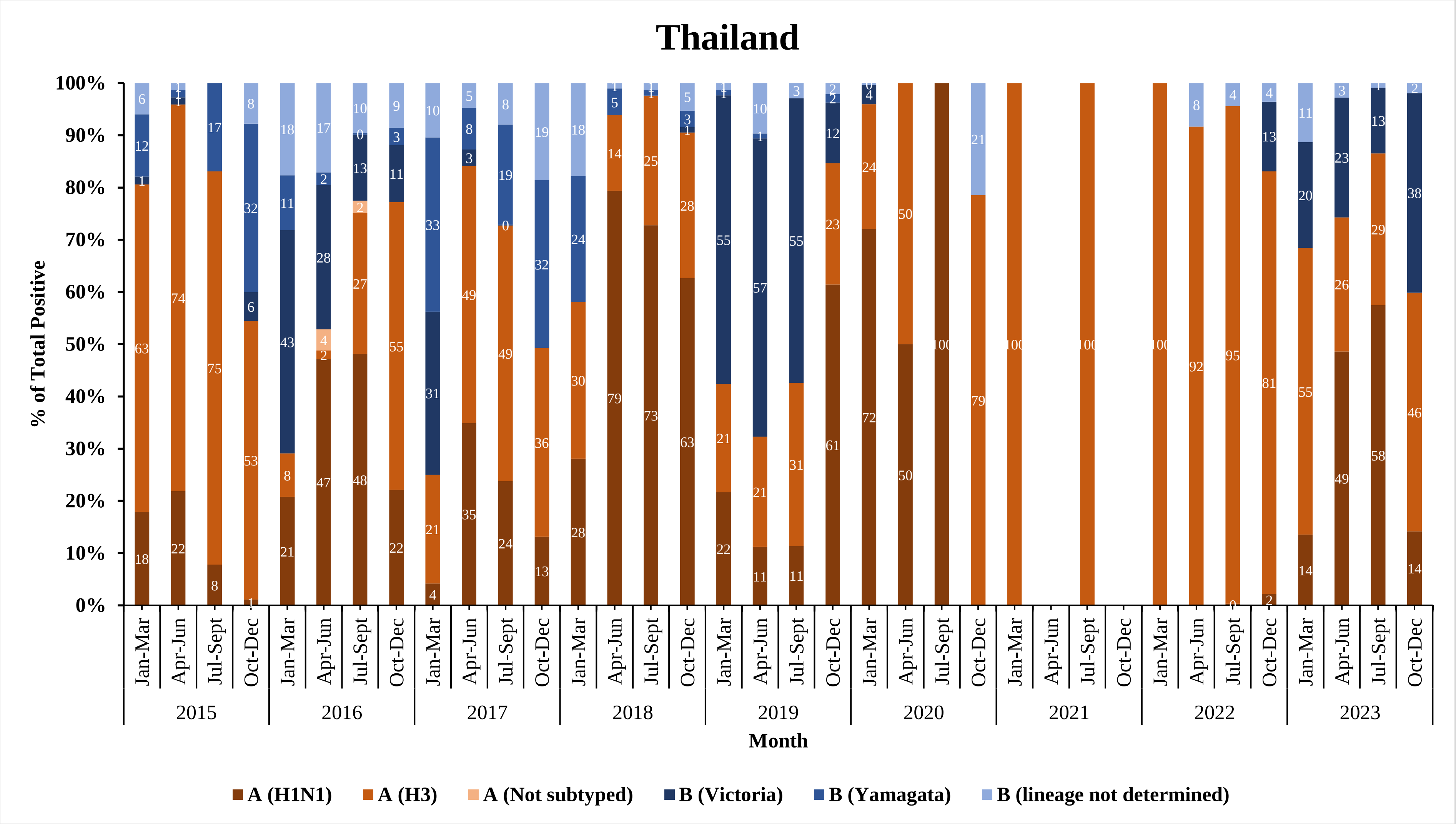

Thailand
% of Total Positive
Month
100%
90%
80%
70%
60%
50%
40%
30%
20%
10%
0%
Jan-Mar
Apr-Jun
Jul-Sept
Oct-Dec
2015
2016
2017
2018
2019
2020
2021
2022
2023
A (H1N1)
A (H3)
A (Not subtyped)
B (Victoria)
B (Yamagata)
B (lineage not determined)

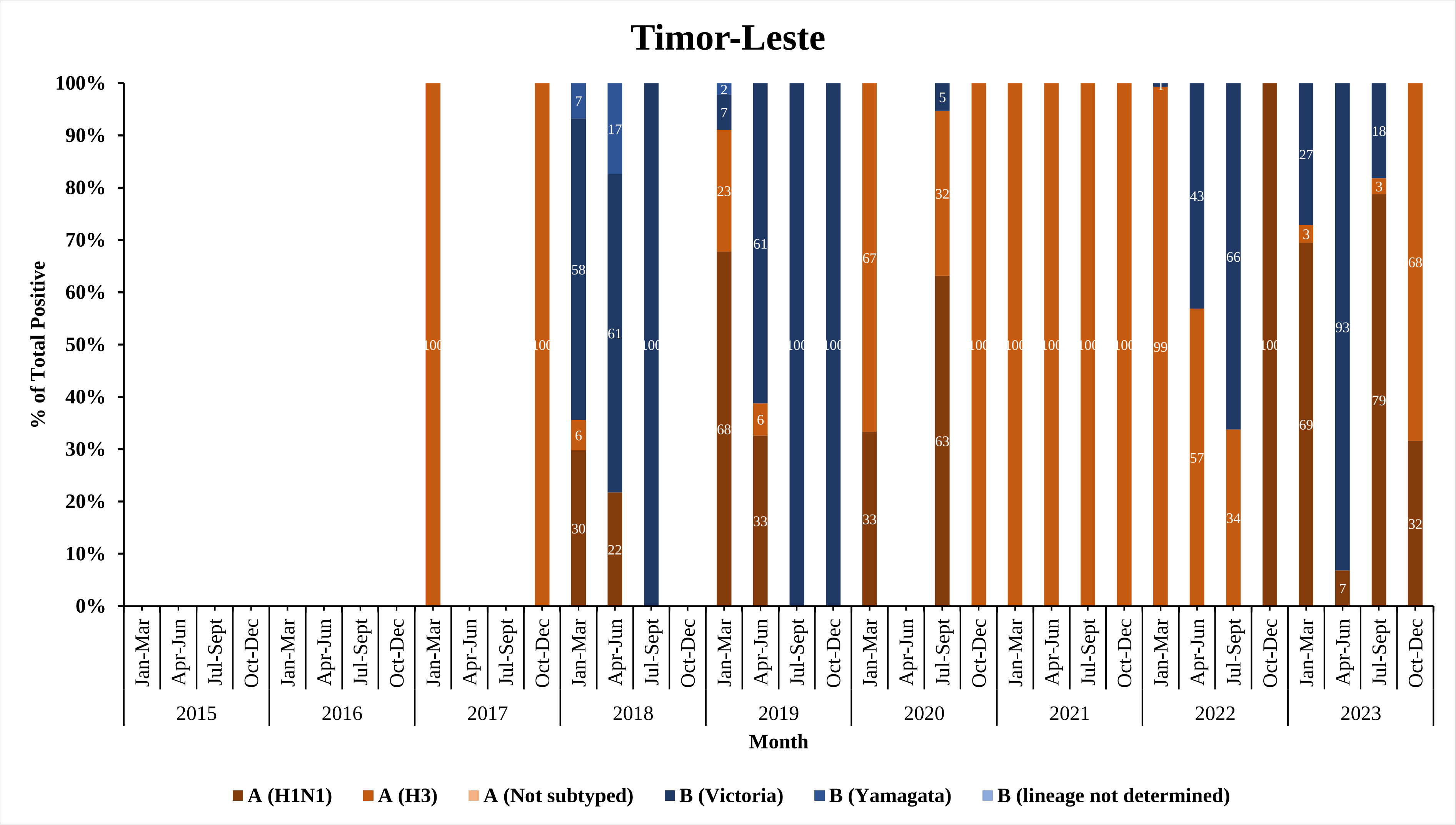

Timor-Leste
% of Total Positive
100%
90%
80%
70%
60%
50%
40%
30%
20%
10%
0%
Jan-Mar
Apr-Jun
Jul-Sept
Oct-Dec
2015
2016
2017
2018
2019
2020
2021
2022
2023
Month
A (H1N1)
A (H3)
A (Not subtyped)
B (Victoria)
B (Yamagata)
B (lineage not determined)
