# Supplementary material for: An analysis of WHO FluNet and FluID influenza surveillance data for South East Asia Region, 2015–2023
Source: PLoS One. 2026 Feb 20;21(2):e0341567. doi: 10.1371/journal.pone.0341567 (PMC12923055; doi:10.1371/journal.pone.0341567)
Supplement: S7 Fig — (PDF) [file pone.0341567.s007.pdf]

S7: Number and the proportion of Influenza-like Illness (ILI) cases per 1000 outpatients and Severe Acute Respiratory Infection (SARI) cases per 100 inpatient, SEAR (2015-2023)

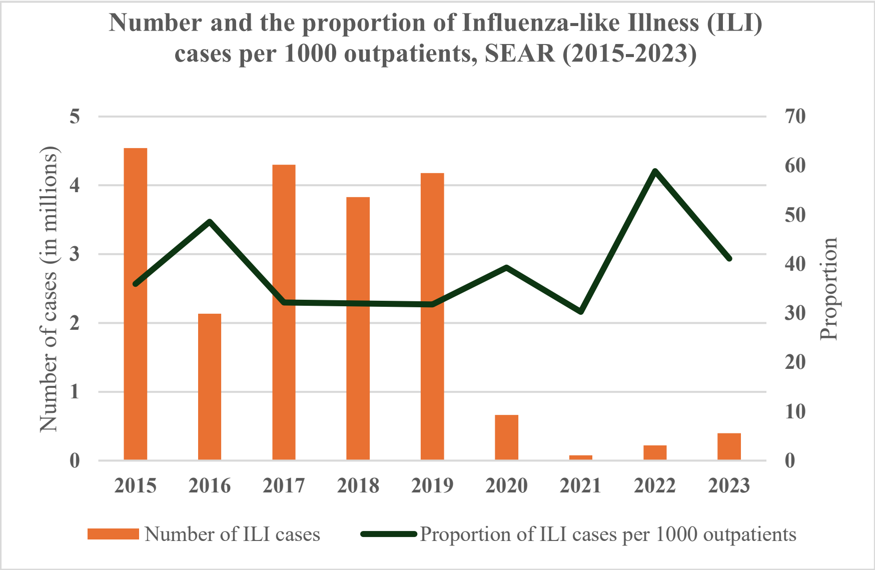

Figure S7.1: Number and the proportion of Influenza-like Illness (ILI) cases per 1000 outpatients, SEAR (2015-2023)

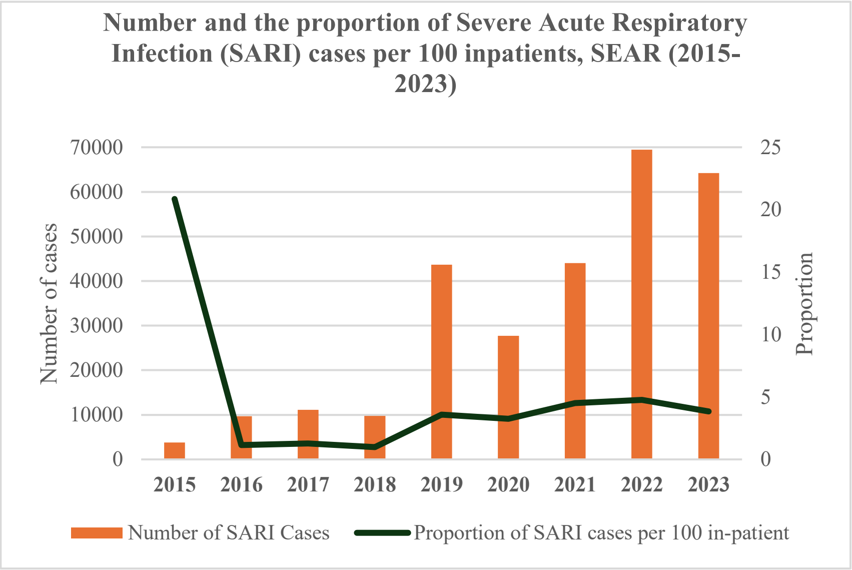

Figure S7.2: Number and the proportion of Severe Acute Respiratory Infection (SARI) cases per 100 inpatients, SEAR (2015-2023)
