# Supplementary material for: An analysis of WHO FluNet and FluID influenza surveillance data for South East Asia Region, 2015–2023
Source: PLoS One. 2026 Feb 20;21(2):e0341567. doi: 10.1371/journal.pone.0341567 (PMC12923055; doi:10.1371/journal.pone.0341567)
Supplement: S8 Table — (PDF) [file pone.0341567.s008.pdf]

S8: Data gaps observed according to the sequence of analysis

Background: Previous studies that have analyzed influenza surveillance data from the WHO FluNet and FluID platforms have noted certain reporting inconsistencies and data completeness issues across member states and years. Similar to these observations, our analysis of WHO South-East Asia Region (SEAR) for the period of 2015-2023 identified certain gaps and inconsistencies that may have affected trend interpretation and cross-country comparability.(1–4) The following observations were made:

1. Inconsistency in reporting of FluNet data

   1a. Study period was from 2015-2023. However, some of the member states started reported from 2016 (Timor-Leste) and 2017 (DPRK).

   1b. In few years, data reporting inconsistencies led to percentages exceeding 100% (where reported processed samples were higher than received samples) which made it difficult to compute the percentage of samples which were actually processed.

   1c. There were several missing values in the data for the total number of samples received (India, Indonesia, Sri Lanka, Thailand and Timor-Leste).

2. Inconsistency in reporting of FluID data

   2a. Only nine SEAR MS reported SARI and ILI cases (excluding India and Myanmar).

   2b. Of the nine MS, only two countries (Bhutan and Indonesia) provided data for the study period (2015-2023).

   2c. Data on SARI deaths were available from only four countries (Bangladesh, Bhutan, Maldives and Nepal) and was also inconsistent throughout the years.

   2d. We excluded data for DPRK for estimating ILI cases per 1000 outpatients and DPRK and Timor Leste for estimating SARI cases per 100 inpatients as there were errors in reporting:

   - Data for Timor-Leste was available for only two years.
   - Number of SARI deaths were more than number of inpatients which made it difficult to compute number of SARI cases per 100 in-patient for both DPRK and Timor-Leste.

   2e. Age categories were reported in the FluID database but they were not standardized across countries or years, with overlapping age groups (e.g., 15-24, 15-29, 15-44 years), making age-wise analysis inconsistent and unreliable.

Below is the given table representing data gaps relevant to each analysis done.

Table S7: Data gaps observed according to the sequence of analysis

S.no.	Analysis	Data inconsistencies in quality and reporting
1.	Influenza data reporting trend in FluNet and FluID databases	1a, 2a, 2b
2.	Influenza virus circulation in WHO SEAR: virological data trends (Source: FluNet)	1b, 1c
3.	Epidemiological data trends for Influenza in SEAR (Source: FluID)	2a, 2b
3.1	ILI and SARI cases according to the hemispheres	2c, 2d, 2e
3.2	ILI and SARI cases according to the WHO Influenza Transmission Zones (ITZ)	2c, 2d, 2e

References:

1. Chen C, Jiang D, Yan D, Pi L, Zhang X, Du Y, et al. The global region-specific epidemiologic characteristics of influenza: World Health Organization FluNet data from 1996 to 2021. Int J Infect Dis. 2023 Apr 1;129:118–24.

2. El Guerche-Séblain C, Caini S, Paget J, Vanhems P, Schellevis F. Epidemiology and timing of seasonal influenza epidemics in the Asia-Pacific region, 2010–2017: implications for influenza vaccination programs. BMC Public Health. 2019 Dec;19(1):1–10.

3. System M of the WPRGIS and R. Epidemiological and Virological Characteristics of Influenza in the Western Pacific Region of the World Health Organization, 2006–2010. PLoS ONE [Internet]. 2012 [cited 2022 Sept 28];7(5). Available from: https://www.ncbi.nlm.nih.gov/pmc/articles/PMC3366627/

4. System M of the WSEARGIS and R. Seasonal influenza surveillance (2009–2017) for pandemic preparedness in the WHO South-East Asia Region. WHO South-East Asia J Public Health. 2020 Jan 1;9(1):55.
